# Supplementary material for: Protocol for an observational cohort study investigating biomarkers predicting seizure recurrence following a first unprovoked seizure in adults
Source: BMJ Open. 2022 Dec 5;12(12):e065390. doi: 10.1136/bmjopen-2022-065390 (PMC9723849; doi:10.1136/bmjopen-2022-065390)
Supplement: Supplementary data [file bmjopen-2022-065390supp001.pdf]

The Walton Centre NHS
NHS Foundation Trust

The Walton Centre NHS Foundation Trust
Lower Lane, Fazakerley
Liverpool, L9 7LJ, UK
Tel: 0151 525 3611
Fax: 0151 529 5500

Dr Guleed Adan
Clinical Research Fellow
2nd Floor, Neurological Science, Clinical Sciences Centre
Aintree University Hospital and The Walton Centre NHS Foundation Trusts
Lower Lane, Liverpool, L9 7LJ
Telephone: 0151 529 5943
Email: guleed@liverpool.ac.uk

PARTICIPANT INFORMATION SHEET FOR PATIENTS (PRAFUS-PIS-V4, 13/03/20)

Research study: Predicting recurrence after first unprovoked seizure (PRAFUS)

You are being invited to take part in a research study. Before you decide it is important for you to understand why the research is being done and what it will involve. Please take time to read the following information carefully. Talk to others about the study if you wish.

- Part 1 tells you the purpose of this study and what will happen to you if you take part.
- Part 2 gives you more detailed information about the conduct of the study.

Ask us if there is anything that is not clear or if you would like more information. Take time to decide whether you wish to take part.

PART 1

What is the purpose of the study?
People who have had a first unprovoked seizure may have an EEG and an MRI scan performed. At the moment, we find that many people with a first seizure often have a normal MRI and EEG. We think this is because we do not yet know the best way to carry out EEG and MRI scanning for people with first unprovoked seizure, and that the current use of scans may not be detailed enough. We have developed some new EEG and MRI scanning methods, and would like to try them out in people who have had a first unprovoked seizure. We hope that these new scans will provide us with a more detailed picture of the structure and function of the brain, which may be very important for people who have a first seizure so that we can better predict those that will go on to have further seizures and therefore develop epilepsy. We are hoping that our EEG and MRI scans can provide information on why some people have further seizures and others do not. We will also be taking blood and saliva samples that will be analysed for novel markers of inflammation that may help to identify whether increased levels of inflammation in the body is linked to the chance of having future seizures.

Why have I been chosen?
You have been chosen because we know you have been recently diagnosed with a first unprovoked seizure.

Do I have to take part?
No. It is entirely up to you to decide whether or not to take part. If you do, you will be given this information sheet to keep and be asked to sign a consent form. You are still free to withdraw at any time and without giving a reason. A decision to withdraw at any time, or a decision not to take part, will not affect the standard of clinical care you receive. If you express an interest in participating in this research, a member of the research team will contact you by your preferred method of correspondence.

What will happen to me if I take part?
We will send you an appointment for the following:

The Walton Centre NHS Foundation Trust
Lower Lane, Fazakerley
Liverpool, L9 7LJ, UK
Tel: 0151 525 3611
Fax: 0151 529 5500

(1) An MRI brain scan at LiMRIC or the Walton Centre. The MRI scan will last about 30 minutes and will involve you lying still and relaxing in the scanner. If your scan takes place at the LiMRIC, you will have some advanced brain scans performed that will involve some extra time to the standard clinical scans that are used currently. This will not compromise your clinical care and your scan will still be analysed and reported by a neuro-radiology consultant at the Walton Centre. Your report will still be returned to the neurology consultant that you are under the care of. A summary of the details of your MRI scan will be sent to you and to your GP as per the Walton Centre's standard clinical practice.

(2) After or before your MRI scan we will take a maximum of three blood tubes (containing nine millilitres each), which is the equivalent of an eggcup full of blood in total. We will also take a small salivary swab from the inside of the cheek. This will take around five minutes. Blood and saliva samples will be analysed and stored in a biobank (freezer) at the LiMRIC for a period of five years or until all of the sample has been used up, whichever is sooner.

(3) Finally, an EEG scan at the Walton Centre, where you will have already been seen by a specialist. This will last around one hour and will involve electrodes being placed on the surface of your head. You will be seated upright, awake and will be relaxed while measurements are taken. The EEG test will likely be on a different day to the MRI scan and blood sample.

There are no more scans or tests after this. A clinical member of the research team will contact you at four different time points after your first seizure: 6 months, 12 months, 18 months and 24 months. This will involve asking basic and brief questions about any further seizures you may have had.

What do I have to do?
All you need to do is sit and relax for the EEG scan and relax and lie still in the MRI scanner. A qualified health care professional will take a sample of your blood and saliva swab before or after your MRI scan.

What are the other possible disadvantages and risks of taking part?
The technique of MRI has been in use in medicine for about 30 years and has shown to be safe. It does not involve any radiation. In some people, there are times when it is not safe to be scanned. For example, in the first three months of pregnancy, or when there are surgical clips inside the brain, or if there is a heart pacemaker or Vagus Nerve Stimulator fitted. Furthermore, the scanner may get warm and noisy, and may not be suitable for sufferers of claustrophobia. However, rest assured, we will discuss with you thoroughly prior to the scan to identify any reasons why you should not have the scan. We will also thoroughly review with you on the day of scanning whether there is any possible risk for you. Earplugs are used to reduce the impact of scanner noise.

What are the possible benefits of taking part?
There may be no direct benefit to you from taking part. We hope that we will gain useful information predicting further seizures in people like yourself from these new scanning techniques, but we cannot be certain about this.

What if there is a problem?
Any complaint about the way you have been dealt with during the visit will be addressed. The detailed information on this is given in Part 2.

Will my taking part in the study be kept confidential?
Yes. All the information about your participation in this study will be kept confidential. The details are included in Part 2.

Contact Details:

The Walton Centre NHS Foundation Trust
Lower Lane, Fazakerley
Liverpool, L9 7LJ, UK
Tel: 0151 525 3611
Fax: 0151 529 5500

For further information, please contact any of the team running this research project:

Dr Guleed Adan
Clinical Research Fellow
2nd Floor, Neurological Science, Clinical Sciences Centre
Aintree University Hospital and The Walton Centre NHS Foundation Trusts
Lower Lane, Liverpool, L9 7LJ
Telephone: 0151 529 5943
Email: guleed@liverpool.ac.uk

Dr. Simon Keller
Senior Lecturer and Researcher in Neuroimaging
2nd Floor, Neurological Science
Clinical Sciences Centre
Aintree University Hospital and The Walton Centre
NHS Foundation Trusts
Lower Lane, Liverpool, L9 7LJ
Telephone: 07795617348
Email: simon.keller@liv.ac.uk

Professor Anthony Marson
Professor of Neurology
2nd Floor, Neurological Science
Clinical Sciences Centre
Aintree University Hospital and The Walton Centre
NHS Foundation Trusts
Lower Lane, Liverpool, L9 7LJ
Telephone: 0151 529 5770
Email: a.g.marson@liv.ac.uk

This completes Part 1 of the Information Sheet.
If the information in Part 1 has interested you and you are considering participation, please continue to read the additional information in Part 2 before making any decision.

The Walton Centre NHS
NHS Foundation Trust

The Walton Centre NHS Foundation Trust
Lower Lane, Fazakerley
Liverpool, L9 7LJ, UK
Tel: 0151 525 3611
Fax: 0151 529 5500

PART 2

What will happen if I don't want to carry on with the study?
You can withdraw from this research study at any time, even while you are having the tests done. This will not affect your treatment in any way. Even if you withdraw from the study, we would still like to use any information we might already have collected.

What if new information becomes available?
It is unlikely that any new information will become available while you are taking part in the study. Because we will be taking pictures of your brain with the MRI scan, occasionally we will have unexpected findings that none of us suspected. The pictures are reviewed by experienced doctors, called neuroradiologists who specialise in looking at pictures of brain. If there are any unexpected findings that need further tests, he/she will write to your GP or specialist. The doctor will then contact you if further tests are required. However, unexpected findings on MRI scans are rare.

What if there is a problem?
If you have a concern about any aspect of this study, you should ask to speak with the researchers who will do their best to answer your questions. If you remain unhappy and wish to complain formally, you can do this through the NHS Complaints Procedure. Details can be obtained from The Walton Centre NHS Foundation Trust:

Patient Experience Team
Sid Watkins Building
The Walton Centre
Lower Lane, Fazakerley, Liverpool, L9 7LJ
Tel: 0151 556 3090/3091
Email: patientexperienceteam@thewaltoncentre.nhs.uk

Further information on official complaints can be found here: https://www.thewaltoncentre.nhs.uk/362/comments-complaints-and-compliments.html

If something does go wrong and you are harmed during the research study there are no special compensation arrangements. If you are harmed and this is due to someone's negligence then you may have grounds for a legal action for compensation against the hospital, but you may have to pay your legal costs. The normal National Health Service complaints mechanisms will still be available to you.

Can I speak to anyone else?
If you would like to speak to someone not part of the research team about MRI scanning in general, please contact:

Professor Graham Kemp
Director, The Liverpool Magnetic Resonance Imaging Centre (LiMRIC)
Pembroke Place, Liverpool, L69 3GE
Telephone: 0151 794 5635; Email: gkemp@liverpool.ac.uk

Supplemental material
BMJ Publishing Group Limited (BMJ) disclaims all liability and responsibility arising from any reliance placed on this supplemental material which has been supplied by the author(s)
BMJ Open

The Walton Centre NHS Foundation Trust
Lower Lane, Fazakerley
Liverpool, L9 7LJ, UK
Tel: 0151 525 3611
Fax: 0151 529 5500

Who is organising and funding the research?
This research is being organised Dr Guleed Adan and is sponsored by The University of Liverpool (see below). A Clinical Research Training Fellowship awarded to Dr Adan, supervised by Prof. Marson and Dr. Keller is funding this research. Neither the research team nor your doctor receives any payment if you take part.

Will my taking part in this study be kept confidential?
Yes. We will collect information about you that could identify you personally (for example, because the information includes your name or date of birth). We will also collect information about you because we believe it might be relevant to understanding the research (e.g. information about your epilepsy, medicine, any previous scans and EEGs, dates and times of seizures near to the MRI scan). This information will be stored in "pseudo-anonymised" form (which means that your name, address and other personal details will be linked with the information we use in the research, but will not be directly accessible during the research) on computers owned by the hospital and on computers owned by the University of Liverpool. These computers will be securely controlled by the research team under the direct responsibility of Dr. Simon Keller and Professor Tony Marson, and no-one outside the team will have access to your information. We will use the information we collect to answer the questions relevant to this research project. Blood and saliva samples will be stored in anonymous form in dedicated research laboratories at the University of Liverpool until samples are used up.

The data that we collect from you (MRI scans, EEG scans, blood and saliva samples) will be kept for future research. All of this data will not include any identifiable information about you. The data may be made available to other researchers that work with the study team, but at no point is any information about who you are indicated or shared.

In the future, it is possible we might have new research questions that could be answered by looking at your information in new ways. We would seek approval from the Research Ethics Committee to use your information for new research projects. If the Research Ethics Committee believed we should contact you again to ask your permission to re-use your information, we will do so. The hospital has a duty to ensure research conducted here is of a high standard and auditors from the hospital may need to review any information we hold about you. The auditors will maintain the highest standards of confidentiality. Procedures for handling, processing, storage and destruction of your data are compliant with the Data Protection Act 1998.

General Data Protection Regulation
The University of Liverpool is the sponsor for this study based in the United Kingdom. We will be using information from you and your medical records in order to undertake this study and will act as the data controller for this study. This means that we are responsible for looking after your information and using it properly. The University of Liverpool will keep identifiable information about you for 20 years after the study has finished if you consent to be contacted about future studies. If you do not consent to being contacted about future relevant studies, your identifiable information will not be kept beyond 5 years after the study has finished.

Your rights to access, change or move your information are limited, as we need to manage your information in specific ways in order for the research to be reliable and accurate. If you withdraw from the study, we will keep the information about you that we have already obtained. To safeguard your rights, we will use the minimum personally-identifiable information possible.

You can find out more about how we use your information by contacting Dr Guleed Adan (or alternative team member). Our Data Protection Officer is Victoria Heath and you can contact them at V.Heath@liverpool.ac.uk.

The University of Liverpool will use your name, NHS number and contact details to contact you about the research study, and make sure that relevant information about the study is recorded for your care, and to

The Walton Centre NHS Foundation Trust
The Walton Centre NHS Foundation Trust
Lower Lane, Fazakerley
Liverpool, L9 7LJ, UK
Tel: 0151 525 3611
Fax: 0151 529 5500

oversee the quality of the study. Individuals from the University of Liverpool and regulatory organisations may look at your medical and research records to check the accuracy of the research study. The Walton Centre will pass these details to the University of Liverpool along with the information collected from you and your medical records. The only people in The University of Liverpool who will have access to information that identifies you will be people who need to contact you to arrange an appointment for the research study investigations or audit the data collection process. The people who analyse the information will not be able to identify you and will not be able to find out your name, NHS number or contact details.

When you agree to take part in a research study, the information about your health and care may be provided to researchers running other research studies in this organisation and in other organisations. These organisations may be universities, NHS organisations or companies involved in health and care research in this country or abroad. Your information will only be used by organisations and researchers to conduct research in accordance with the UK Policy Framework for Health and Social Care Research (https://www.hra.nhs.uk/planning-and-improving-research/policies-standards-legislation/uk-policy-framework-health-social-care-research/).

This information will not identify you and will not be combined with other information in a way that could identify you. The information will only be used for the purpose of health and care research and cannot be used to contact you or to affect your care. It will not be used to make decisions about future services available to you, such as insurance.

Involvement of your doctor
The doctor looking after you in the hospital will be aware of your participation in this research study.

What will happen to the results of the research study?
The scientific results of this research study will be published in scientific and medical journals and may be discussed at scientific meetings. You will not be personally identified in any way.

Who has reviewed the study?
North East - Tyne and Wear South Research Ethics Committee has reviewed this study and given a favourable ethical opinion for this research.

You will be given a copy of the information sheet and a copy of your signed consent form to keep.

Thank you for considering taking part in this research project and thank you for taking the time to read the information sheets.
