# Supplementary material for: Protocol for an observational cohort study investigating biomarkers predicting seizure recurrence following a first unprovoked seizure in adults
Source: BMJ Open. 2022 Dec 5;12(12):e065390. doi: 10.1136/bmjopen-2022-065390 (PMC9723849; doi:10.1136/bmjopen-2022-065390)
Supplement: Supplementary data [file bmjopen-2022-065390supp002.pdf]

The Walton Centre NHS
NHS Foundation Trust

The Walton Centre NHS Foundation Trust
Lower Lane, Fazakerley
Liverpool, L9 7LJ, UK
Tel: 0151 525 3611
Fax: 0151 529 5500

CONSENT FORM

Short title of Project: Predicting recurrence after first unprovoked seizure (PRAFUS)
Version 2=3: PRAFUS-CON-V3; 13/03/2020
IRAS Project ID: 279362**;** Research ethics committee ID:

Name of Chief Investigator: Prof. Tony Marson

	**Please initial box**
1. I confirm that I have read and understand the participant information sheet dated 13/03/20 (Version 4: **PRAFUS-PIS-V4**) for the above study. I have had the opportunity to consider the information, ask questions and have had these answered satisfactorily.	☐
2. I understand that my participation is voluntary and that I am free to withdraw at any time, without giving any reason, without my medical care or legal rights being affected.	☐
3. I understand that relevant sections of any of my medical notes and data collected during the study may be looked at by responsible individuals from regulatory authorities or from the NHS Trust, where it is relevant to my taking part in this research. I give permission for these individuals to have access to my records.	☐
4. I agree for a blood and saliva sample to be taken and used for future research. I understand that blood and saliva samples will be stored in anonymous form in dedicated research laboratories at the University of Liverpool for a period of five years or until samples are depleted, whichever is sooner.	☐
5. I agree for the research team to share anonymised data collected in this study with other researchers; I understand that I will not be identified in anyway.	☐
6. I agree to be contacted by a clinical member of the research team at 6, 12, 18 and 24 months after my MRI scan by telephone who will ask me brief questions about my seizures and medication.	☐
7. I agree that I may be allocated to have advanced MRI brain scans to be performed at the University of Liverpool, which will include some additional scanning (sequences) to the standard clinical scans that would have otherwise been performed at the Walton Centre. I understand that my clinical care will not be compromised should I agree to this.	☐

When completed, 1 for patient; 1 for researcher site file; 1 (original) to be kept in medical notes

The Walton Centre NHS
NHS Foundation Trust

The Walton Centre NHS Foundation Trust
Lower Lane, Fazakerley
Liverpool, L9 7LJ, UK
Tel: 0151 525 3611
Fax: 0151 529 5500

8. I agree to be contacted in the future should another scanning study become available. ☐

Preferred method of contact:

______________________________ Address
______________________________ Email

______________________________ Home telephone
______________________________ Mobile telephone

9. I agree to take part in the above study. ☐

Name of Participant	Date	Signature
Name of legal guardian or parent ***(if participant is under 18)***	Date	Signature
Name of Person taking consent (if different from researcher)	Date	Signature
Researcher	Date	Signature

When completed, 1 for patient; 1 for researcher site file; 1 (original) to be kept in medical notes
