# Supplementary material for: Protocol for an observational cohort study investigating biomarkers predicting seizure recurrence following a first unprovoked seizure in adults
Source: BMJ Open. 2022 Dec 5;12(12):e065390. doi: 10.1136/bmjopen-2022-065390 (PMC9723849; doi:10.1136/bmjopen-2022-065390)
Supplement: Author's manuscript [file bmjopen-2022-065390.draft_revisions.pdf]

BMJ Open

BMJ Open is committed to open peer review. As part of this commitment we make the peer review history of every article we publish publicly available.

When an article is published we post the peer reviewers' comments and the authors' responses online. We also post the versions of the paper that were used during peer review. These are the versions that the peer review comments apply to.

The versions of the paper that follow are the versions that were submitted during the peer review process. They are not the versions of record or the final published versions. They should not be cited or distributed as the published version of this manuscript.

BMJ Open is an open access journal and the full, final, typeset and author-corrected version of record of the manuscript is available on our site with no access controls, subscription charges or pay-per-view fees (http://bmjopen.bmj.com).

If you have any questions on BMJ Open's open peer review process please email info.bmjopen@bmj.com

BMJ Open

A protocol for an observational cohort study investigating biomarkers predicting seizure recurrence following a first unprovoked seizure in adults.

Journal:	*BMJ Open*
Manuscript ID	bmjopen-2022-065390
Article Type:	Protocol
Date Submitted by the Author:	19-Jul-2022
Complete List of Authors:	Adan, Guleed; University of Liverpool, Department of Pharmacology and Therapeutics; The Walton Centre NHS Foundation Trust<br>de Bézenac, Christophe; University of Liverpool, Molecular and Clinical Pharmacology<br>Bonnett, Laura; University of Liverpool Department of Biostatistics<br>Pridgeon, Michael; The Walton Centre NHS Foundation Trust<br>Biswas, Shubhabrata; The Walton Centre NHS Foundation Trust<br>Das, Kumar; The Walton Centre NHS Foundation Trust<br>Richardson, Mark P.; King's College London Institute of Psychiatry Psychology and Neuroscience, Department of Basic and Clinical Neuroscience<br>Laiou, Petroula; King's College London Institute of Psychiatry Psychology and Neuroscience, Department of Basic and Clinical Neuroscience<br>Keller, Simon S.; University of Liverpool, Department of Pharmacology and Therapeutics; The Walton Centre NHS Foundation Trust<br>Marson, Tony; University of Liverpool, Department of Pharmacology and Therapeutics; The Walton Centre NHS Foundation Trust
Keywords:	Epilepsy < NEUROLOGY, NEUROPHYSIOLOGY, Magnetic resonance imaging < RADIOLOGY & IMAGING

SCHOLARONE™
Manuscripts

A protocol for an observational cohort study investigating biomarkers predicting seizure recurrence following a first unprovoked seizure in adults.

Guleed Adan[1,2,],Christophe de Bézenac[1,2],Laura Bonnett[3],Michael Pridgeon[2], Shubhabrata Biswas[2], Kumar Das[2], Mark P. Richardson[4], Petroula Laiou[4],Simon S. Keller[1,2] Tony Marson[1,2]

1. Department of Pharmacology and Therapeutics, Institute of Systems, Molecular and Integrative Biology, University of Liverpool.

2. The Walton Centre NHS Foundation Trust, Lower Lane, Liverpool, L9 7LJ, United Kingdom.

3. Department of Biostatistics, University of Liverpool, Liverpool, L69 3BX, United Kingdom.

4. Department of Basic and Clinical Neuroscience, Institute of Psychiatry, Psychology and Neuroscience, King's College London, London, UK

Trial registration:

NIHR Clinical Research Network's (CRN) Central Portfolio Management System (CPMS) - 44976

World Health Organization Trial Registration Data Set:

Data category	Information
Primary registry and trial identifying number	NIHR CRN CPMS 44976
Date of registration in primary registry	11th March, 2020
Secondary identifying numbers	IRAS 279362, UoL0015106107, REC reference 20/NE/0078
Source(s) of monetary or material support	Association of British Neurologists and Guarantors of Brain
Primary sponsor	Prof Neil French, University of Liverpool [sponsor@liv.ac.uk]
Contact for public queries	Dr Guleed Adan [guleed@liv.ac.uk]
Contact for scientific queries	Dr Guleed Adan [guleed@liv.ac.uk]<br>Department of Pharmacology and Therapeutics, University of Liverpool
Public title	Predicting recurrence after a first unprovoked seizure (PRAFUS)
Scientific title	Predicting recurrence after a first unprovoked seizure — a prospective observational cohort study
Countries of recruitment	England, UK
Health condition(s) or problem(s) studied	First unprovoked seizure, epilepsy, seizure recurrence
Intervention(s)	None.
Key inclusion and exclusion criteria	Ages eligible for study: ≥18 years<br>Sexes eligible for study: both<br>Accepts healthy volunteers: no

For peer review only

Data category	Information
	Inclusion criteria: adult patient (≥ 18 years), diagnosed with a first unprovoked seizure, maximum of 8 weeks since first seizure
	Exclusion criteria: provoked seizures, dissociative seizures, progressive or inflammatory neurological disorder, previous neurosurgery
Study type	Observational
	Primary purpose: development of a predictive prognostic model
Date of first enrolment	17th August 2020.
Target sample size	100
Primary outcome(s)	Time to seizure recurrence (follow up time: 24 months)

For peer review only

Date and protocol version identifier:

Version 1: 23/09/2019, University of Liverpool.
Version 2: 02/10/2019, University of Liverpool.
Version 3: 31/10/2019, University of Liverpool.
Version 4: 13/11/2019, University of Liverpool.
Version 5: 05/12/2019, University of Liverpool.
Version 6: 17/01/2020, University of Liverpool.
Version 7: 09/03/2020, University of Liverpool.
Version 8: 13/03/2020, University of Liverpool.
Version 9: 29/05/2020, University of Liverpool.

Correspondence to:

Dr. Guleed Adan
University of Liverpool
Dept of Neurological Sciences
Clinical Sciences Centre
Lower Lane
Liverpool
L9 7LJ
UK

Guleed.adan@liverpool.ac.uk

Tel: +44 (0)7783844562

For peer review only - http://bmjopen.bmj.com/site/about/guidelines.xhtml

ABSTRACT

Introduction:

A first unprovoked seizure is a common presentation, reliably identifying those that will have recurrent seizures is a challenge. This study will be the first to explore the combined utility of serum biomarkers, quantitative EEG and quantitative MRI to predict seizure recurrence. This will inform patient stratification for counselling and the inclusion of high-risk patients in clinical trials of disease modifying agents in early epilepsy.

Methods and analysis: 100 patients with first unprovoked seizure will be recruited from a tertiary neuroscience centre and baseline assessments will include structural MRI, EEG, and a blood sample. As part of a nested pilot study, a subset of 40 patients will have advanced MRI sequences performed that are usually reserved for patients with refractory chronic epilepsy. The remaining 60 patients will have standard clinical MRI sequences. Patients will be followed up every 6 months for a 24-month period to assess seizure recurrence. Connectivity and network-based analyses of EEG and MRI data will be carried out and examined in relation to seizure recurrence. Patient outcomes will also be investigated with respect to analysis of high mobility group box-1 (HMGB1) from blood serum samples.

Ethics and dissemination: This study was approved by North East – Tyne & Wear South Research Ethics Committee (20/NE/0078) and funded by an Association of British Neurologists (ABN) and Guarantors of Brain (GoB) clinical research training fellowship. Findings will be presented at national and international meetings published in peer-reviewed journals.

Keywords: First unprovoked seizure, Prognostic modelling, Brain connectivity, Quantitative MRI, Biomarker

Strengths and Limitations

- This will be the first study to prospectively investigate how brain structural and physiological architecture and connectivity in adults influences seizure recurrence following a first unprovoked seizure.
- The study is expected to provide insights into the biology underlying epileptogenesis, and to lead to the development of prognostic markers of seizure recurrence following a first unprovoked seizure.
- Expected recruitment has been based on records of past diagnosis and while the study is expected to recruit well, unexpected under-recruitment is possible and would be a barrier to timely completion.
- A second potential limitation of this study is the potential for participant attrition and loss of patient follow up at multiple points over 24 months; missing data could impact on the validity of study conclusions.

For peer review only - http://bmjopen.bmj.com/site/about/guidelines.xhtml

INTRODUCTION

Background and rationale

A first unprovoked seizure is a common presentation; the annual incidence is between 50 and 70 per 100,000 (1). At least 10% of the population will have at least one seizure (2) and approximately 50% will have a recurrence (3). A major challenge is to reliably identify those that will have recurring seizures, to better inform treatment decisions and counselling (4). This will be increasingly important as we try to develop disease-modifying treatments. One paradigm will be to test new and repurposed treatments early in the disease course, using a design similar to the Multi-centre Study of Early Epilepsy and Single Seizures (MESS) (5). Efficient trial designs will require the recruitment of people at high risk of seizure recurrence and this study will aid the development of EEG, MRI and blood biomarkers to do so.

Various investigations are used in the diagnostic work up of patients with first unprovoked seizures including electroencephalogram (EEG) and neuroimaging, which have been shown to have some prognostic value, but current prognostic models lack precision to reliably stratify patients. Prognostic models of data from the Multi-centre Study of Early Epilepsy and Single Seizures (MESS) study (6,7) identified epileptiform EEG abnormality, neurological deficit and abnormal magnetic resonance imaging (MRI) as significant prognostic factors. For these models, EEG and MRI were simply classified as normal, abnormal or 'non-specific'. It is now possible to use advanced quantitative approaches to analyse EEG and MRI and use them as continuous measures of neurophysiological function and anatomical variation, as proposed in this study.

In this study we will investigate emerging epilepsy biomarkers in first seizure populations. We already know that patients with an MRI lesion have a higher risk of recurrence (6, however, advanced quantitative MRI analysis has never been used in this population. These methods have been demonstrated by the Liverpool Epilepsy Research Group to be effective in predicting seizure outcomes in pre-surgical patients with temporal lobe epilepsy (8,9). High-mobility group box 1 (HMGB1) is a key neuro-inflammatory mediator in epilepsy. Increased levels of expression of HGMB1 have been shown by the Liverpool group to be associated with increased seizure frequency in newly diagnosed epilepsy (10). Computational analysis of resting-state EEG has been shown to reliably differentiate between cases of idiopathic generalised epilepsy and healthy controls (11).

Trial design

A prospective, observational cohort study that includes a nested exploratory study;

1. a prospective study in which those EEG and MRI biomarkers will be further refined and validated. Inflammatory biomarkers in the blood and saliva will also be assessed.
2. a nested exploratory study in which the utility of advanced quantitative MRI biomarkers in patients with a first unprovoked seizure will be assessed.

Study setting

Participants will be recruited from a tertiary academic neuroscience centre in England.

Rationale for study

Hypothesis

Following a first unprovoked seizure, patients at high risk of a recurrence can be identified using a combination of EEG, MRI, blood serum inflammatory biomarkers and clinical factors.

Overall aims

The overall aim is to undertake a multi-modal investigation of brain structure, connectivity and inflammation in adults with a first unprovoked seizure. The proposed project will provide new insights into the biology underlying first unprovoked seizures in humans whilst also allowing us to develop prognostic markers of seizure recurrence. This research will take place in the context of collaboration between researchers with an internationally respected reputation for research in epilepsy, and in an environment with demonstrated excellence in recruitment of patients with both first seizure and newly diagnosed epilepsy into research studies and clinical trials.

Prospective sub-study

For peer review only - http://bmjopen.bmj.com/site/about/guidelines.xhtml

Objectives:

1. To externally validate the prognostic markers of seizure recurrence following first unprovoked seizure that have been developed in a retrospective study
2. To identify blood and salivary biomarkers of seizure recurrence after first unprovoked seizure
3. To identify, in a subset of 40 patients, potential biomarkers of seizure recurrence from advanced quantitative image analysis*

NB: The methods described in this protocol apply to both the prospective study and the exploratory study unless otherwise stated.

METHODS AND ANALYSIS

Outcomes

The primary outcome event being studied is seizure recurrence following a first unprovoked seizure. We propose to use a multivariable prognostic model which evaluates the utility of multi-modal biomarkers of seizure recurrence using a time-to-event-outcome, with the event of interest being seizure recurrence. Seizure recurrence will be identified at follow-up intervals of 6, 12, 18 and 24 months.

Population

100 patients with a first unprovoked seizure will be recruited from "first seizure clinics" at the WCFT. Suitable patients will be identified by treating clinicians directly from clinic and also from the electronic patient records. A summary of the recruitment process is shown in Figure 1 and highlighted in more detail in the following section. Only patients that satisfy the inclusion and exclusion criteria below will be recruited into the study.

(Insert Figure 1 here)

Inclusion criteria:

- Aged over 16 years*
- Diagnosis of a first unprovoked seizure made at WCFT clinic
- Maximum of eight weeks since first unprovoked seizure

**recruited participants aged between 16-18 years will have appropriate parental consent sought in addition to their own, additional space for appropriate parental signatures will be available on the standard consent form used in the study to allow for this eventuality.*

Exclusion criteria:

- Provoked seizures (e.g. alcohol or drug induced)
- Non-epileptic seizures
- Acute symptomatic seizures (e.g. acute brain haemorrhage or brain injury)
- Known progressive neurological disease (e.g. brain tumour, Alzheimer's disease).
- Known inflammatory neurological condition (specifically multiple sclerosis or sarcoidosis)
- Previous neurosurgery
- None ambulatory patients with known significant issues with mobility which impairs ability to independently transfer onto MRI scanner e.g. hoist transfer dependent

For peer review only - http://bmjopen.bmj.com/site/about/guidelines.xhtml

For peer review only

- Significant medical co-morbidity e.g. pre-existing severe cardiovascular or respiratory disease that would make them unsuitable for the prolonged supine positioning required for MRI scanning
- Inability to understand written or spoken English

Withdrawal criteria:

- Participants may withdraw their participation in this study by contacting the CI or a member of the study research team at any time.

Recruitment via the outpatient/telemedicine clinic:

A clinical member of the wider research team (i.e. consultant neurologist, epilepsy nurse or neurology specialist trainee) will enquire whether eligible patients would be interested in participating in this study at the time of consultation during outpatient/telemedicine clinics.

Recruitment via the electronic patient record system:

Patients who have been coded as having a first unprovoked seizure from the WCFT electronic patient records will be assessed according to the eligibility criteria mentioned previously. Suitable patients will then be contacted by telephone by a clinical member of the epilepsy team at the Walton Centre to enquire whether eligible patients would be interested in participating in this study.

Procedures/Assessments

Summary of the procedures for each participant (Table 1):

Table 1. Summary of the procedures for each participant:

Procedure	Location	Duration	Number of examinations
1.MRI	*LiMRIC, UoL or WCFT	1 hour, including safety examination and set up	1
2.Blood extraction and saliva collection	*LiMRIC, UoL or WCFT	5 minutes	1
3.EEG	Neurophysiology, WCFT	1 hour, including set up	1
4.Telephone questionnaire	Home	5 minutes	4 (6, 12, 18 and 24 months after index event)

**Patients in the exploratory study within the prospective cohort will have their imaging, saliva and blood sampling performed at the LiMRIC, UoL only instead of the WCFT.*

Study timeline

For peer review only - http://bmjopen.bmj.com/site/about/guidelines.xhtml

The proposed study will last three years and will be split into three phases which are outlined below. We require a recruitment period long enough to recruit a sufficient number of patients with first unprovoked seizure and a follow up period long enough to establish likely seizure recurrence.

Phase 1 (Ph1; *month 1-3*) is an initial *3-month* period dedicated to project set-up and optimisation of the MRI protocol. MRI optimisation will include technical development MRI scanning of phantoms and human volunteers to ensure the MRI sequences are adequate for the study. Necessary sponsorship and ethical approval will be sought during phase 1.

Phase 2 (Ph2; *month 4-24*) is a *20-month* period that includes participant recruitment, baseline clinical data collection, MRI, EEG, blood and saliva acquisition for all recruited participants.

Phase 3 (Ph3; *month 10-34)* is a 24-month patient follow-up period during which time all seizure recurrence information will be recorded by telephone by at 6,12,18 and 24 months after the index event.

Sample size calculation

We propose to use a multivariable prognostic model using time-to-event-outcome with the event of interest being seizure recurrence. With multiple variables of equal interest, development of a 'standard' sample size formulae is problematic. Therefore, we propose to use an events per variable calculation. The most often cited recommendation is the rule of '10 events per variable (EPV)' (12). We will recruit 100 patients therefore, with an assumed 2 year seizure recurrence rate of 51% (13), we would be able to include up to 5 predictor levels.

40 of the 100 patients recruited will have additional advanced MRI sequences performed as part of the nested exploratory study. A sample size of 40 was chosen largely to cost limitations and given that it would satisfy the sample size flat rule of having at least 30 participants (14).

Data acquisition*

In total, we will prospectively perform clinical MRI scans, routine EEG, saliva and blood sample investigations in all of the 100 patients recruited prospectively. A table detailing data collection at the various timepoints of the study is presented below (Table 2).

Table 2. Study Timepoints

		Follow Up Schedule			
Procedures	Baseline (T0)[1]	T0+6 months	T0+12 months	T0+18 months	T0+24months
Signed Consent Form	X				
Assessment of Eligibility Criteria	X				
Contact details	X				
Review of Medical History and demographics including:<br>• Age<br>• Gender	X				

For peer review only - http://bmjopen.bmj.com/site/about/guidelines.xhtml

		Follow Up Schedule			
Procedures	Baseline (T0)[1]	T0+6 months	T0+12 months	T0+18 months	T0+24months
• Seizure type<br>• Neurological deficit<br>• Febrile seizures<br>• Family history of epilepsy					
Investigations (EEG, MRI, blood and saliva sampling)	X				
Review of seizure occurrence by telephone		X	X	X	X

*In addition to the standard clinical MRI sequences which all 100 participants will have performed, advanced quantitative MRI scanning will be performed at the LiMRIC main campus for 40 patients that are part of the cohort in the exploratory sub-study outlined previously.

All EEG data collection will take place at the WCFT, in the department of neurophysiology.

MRI

All 100 participants recruited will have clinical MRI scans performed which will include the following sequences:

1. Conventional 2D T2-weighted fast spin echo and fast Fluid Attenuated Inversion Recovery scans, for incidental findings screening, and detection of gross pathology (together with localizer ~10 minutes)

2. 3D T1-weighted MPRAGE scan with isotropic voxel size of 1 mm x 1 mm x 1mm (~10 minutes);

As part of the exploratory nested study looking at the predictive utility of advanced imaging in first seizure patients, 40 patients will have advanced MRI scans performed (max 30 minutes) and will consist of:

1. Conventional 2D T2-weighted fast spin echo and fast Fluid Attenuated Inversion Recovery scans, for incidental findings screening, and detection of gross pathology (together with localizer ~10 minutes)

2. 3D T1-weighted MPRAGE scan with isotropic voxel size of 1 mm x 1 mm x 1mm (~10 minutes);

3. high resolution diffusion kurtosis imaging (DKI) sequence with at least 60 isotropically distributed gradient directions, three b values (b=0, 1000 and 2000) and maximum voxel size of 2 mm x 2 mm x 2mm (~10 minutes).

MRI Safety Criteria

All participants who are having an MRI scan as part of the study will have a completed MRI safety screening as a pre-requisite.

For peer review only - http://bmjopen.bmj.com/site/about/guidelines.xhtml

All participants will be examined by a radiographer and will complete a safety checklist that is designed to identify whether a participant has internal bodily metal, which could pose a hazard during MRI scanning. All removable bodily metal will be removed before scanning.

Blood extraction

All 100 patients will have blood collected for analysis in a Lithium-Heparin bottles or serum separator tubes (9mls). A maximum of 27 milliliters of blood (3 x 9ml vials) will be obtained from each participant. Samples will be obtained by a healthcare professional trained in phlebotomy. A standard operating procedure for blood sampling including aseptic technique will be utilised by all practitioners involved in the study. Blood samples will be centrifuged within 15 minutes of collection or stored overnight at 4°c for centrifuge the following day. 250 μl aliquots will then be transferred to appropriate tubes and stored at approximately -80 degrees Celsius prior to bioanalysis. This process is identical to other studies in the group running in parallel with REC approval (REC reference 17/NW/0342 and 19/NW/0384).

EEG

All 100 patients will undergo a conventional clinical EEG, using 19 channels in 10-20 arrangement, at the Department of Neurophysiology at the WCFT. Participant visiting time will last approximately one hour.

Saliva

All 100 patients will have samples of unstimulated saliva collected by soaking a sponge swab in the mouth of each participant until the swab is saturated with saliva. The swab will be inserted into a collection tube. In the laboratory, the saliva sample will then be collected into an Eppendorf tube by squeezing the saturated swab using a syringe. The sample will be stored at -80 C freezer until assay.

Data analysis

Clinical MRI

Analysis of clinical MRI data will be performed in all 100 participants recruited. We will perform morphometric analysis of subcortical structures which we know are implicated in epileptic seizures. Analysis of the data will involve using stereology in conjunction with point counting (16,17) and an automated method of volumetry for 3DT1-weighted MRI data (18) to estimate the volume of the hippocampus, amygdala, thalamus, and basal ganglia in all patients.

Advanced MRI*

**analysis of advanced MRI data will only be performed in the 40 participants that are included in the exploratory sub-study*

(1) Thalamocortical. Preliminary data from our group has indicated that patients with newly diagnosed epilepsy who continue to experience seizures despite AED therapy have diffusion ketosis imaging (DKI) alterations of thalamic projections; we will apply the same DKI approaches in our prospective study. Mean DKI values will be obtained from spatially co-registered regions-of-interest (principally thalamocortical regions) in standard space. We will also apply diffusion and resting-state functional MRI independent component analysis techniques using FSL's MELODIC toolbox (http://fsl.fmrib.ox.ac.uk/fsl/fslwiki/MELODIC) and in-house Matlab scripts to identify abnormal structural and functional thalamocortical connectivity in patients relative to controls (19,20,21). (2) White matter tracts. Our recent publications have indicated that analysis of white matter tract diffusion has significance for predicting postsurgical seizure outcome in patients with chronic focal epilepsy and that DKI is more sensitive to tract pathology than diffusion tensor imaging in epilepsy (8,9,22). As white matter tracts constitute the structural connections within brain networks,

For peer review only - http://bmjopen.bmj.com/site/about/guidelines.xhtml

we will determine DKI properties along the length of multi-lobar white matter tract bundles, using our recently reported methods (22, 23). (3) Connectome. The development of whole brain connectomes from diffusion MRI data have led to successful data-driven approaches to predict surgical responsiveness in patients with refractory focal epilepsy from members of our group (24,25,26,27). Connectome approaches also support the association between postoperative seizure control and thalamocortical connectivity (24). Similar methods have been applied to resting-state functional MRI data to model functional connectome alterations in chronic focal epilepsy (28). As per our recent connectomic studies, whole brain structural connectomes will be generated for each participant using T1-weighted and DKI data. T1-weighted data will be parcellated into multiple regions of interest (ROI; or nodes) using Freesurfer software (http://freesurfer.net). Structural connectivity between nodes will be determined using FSL's diffusion toolbox (http://fsl.fmrib.ox.ac.uk/fsl/fslwiki/FDT) for probabilistic fibre tracking applied to diffusion MRI. Structural connectomes will be generated using the Connectome Mapping Toolkit (http://www.connectome.ch). We will use graph theory to determine global and regional network configuration. Global network "small worldness" will be assessed, representing the ratio between average nodal clustering coefficients and as network efficiency. Regional clustering coefficient, efficiency and centrality will also be calculated for key brain areas associated with seizure onset and propagation, such as thalamocortical and limbic networks.

EEG

The resting-state EEG activity of all 100 patients recruited will be identified by a trained clinical EEG professional. Nodes in EEG networks will be defined as electrodes, and a range of measures of interdependence between electrodes will be explored. We will apply computer models of network dynamics to resting-state EEG data (11).

Blood samples

Samples from all 100 patients recruited will be analysed for inflammatory markers, namely HMGB1. Inflammatory marker and HMGB1 expression analysis will be undertaken by ELISA.

Saliva

Saliva samples will be analysed from all 100 patients to assess for concentration levels of circulating inflammatory markers including cytokine profile

Statistical analysis

To explore the utility of imaging, EEG and circulating inflammatory markers a series of univariable Cox regression models and a multivariable Cox regression model will be fitted for the outcome of time to next seizure following the index event, building on previous models developed with the MESS data (6). For model development at least 10 events per candidate predictor variable is advocated (12). With 100 patients and an assumed 2 year seizure recurrence rate of 51% (13), we would be able to include up to 5 predictor levels. Backward selection, using all candidate factors (imaging, EEG and HMGB1),according to Akaike's Information Criterion (15) will be used to determine the parsimonious model. Bootstrap resampling with 1000 replications will be used to adjust the developed model for optimism. Accuracy of model predictions will be explored using discrimination (Harrell's c-statistic) and calibration (calibration slope) both in the data used for model development, and in the prospective data, which will be used for external validation.

Patient and Public Involvement

The development of the research question and outcome measures used in this study have been informed by close collaboration with local and national epilepsy charities. Patient groups were able to be consulted on their priorities, experience and preferences. Patients and their families through the Mersey Regional Epilepsy Association are involved with all research activity by our group. Patients are involved in the recruitment and conduct of the whole study. Results will be disseminated once available through epilepsy research websites, social media channels and mailing lists.

For peer review only - http://bmjopen.bmj.com/site/about/guidelines.xhtml

ETHICS AND DISSEMINATION

Ethical Approval

The Chief Investigator will obtain approval from the appropriate Research Ethics Committee (REC) through IRAS. The Chief Investigator will ensure a copy of the Trust R&D approval letter is available before accepting participants into the study. The study will be conducted in accordance with the recommendations for physicians involved in research on human subjects adopted by the 18th World Medical Assembly, Helsinki 1964 and later revisions.

Human Material

Blood and saliva samples will be taken with appropriate informed consent from all 100 of the prospectively recruited participants.

Samples will be taken in the Walton Centre NHS Foundation Trust, main outpatient building, on the day of MRI scan for the 60 participants who are having clinical MRI sequences only performed. For the 40 participants who will be having advanced brain imaging performed in addition to standard clinical MRI sequences, they will have their samples taken at the LiMRIC on the day of their MRI scan.

Blood and saliva samples will be stored in the Liverpool University Biobank (LUB) freezer room, which is housed in the Research Technology building within the LiMRIC. All samples will be stored and accessed in line with LUB standard operating procedures (SOP).

Samples of human material will be stored and archived in the Liverpool University Biobank for a period of five years following initiation of the study, after which time they will be safely and appropriately destroyed in line with standard practice. The five-year period was chosen to allow a reasonable time frame for further analysis, following ethical clearance, after completion of the study which is expected to be within three years. Explicit consent will be taken from patients for the prolonged storage of this material beyond the study end date, the material will be composed of unused serum and saliva samples in case of further unspecified analysis pending ethical approval. In line with LUB SOP, the University of Liverpool will act as the custodian of the stored serum and saliva samples and explicit consent of the CI will be sought for any application to use the samples for any other purpose other than for those of the study outlined in this protocol.

Anonymity and data governance

All EEG and MRI data will be anonymised prior to being exported from the WCFT and LiMRIC respectively. Personal information will not be identifiable from the data. Names will be replaced with study ID numbers (PRAFUS001, PRAFUS002 etc), which can be backtracked to participant details using a key that is located with the chief investigator, AM, who is part of the primary care team.

Digital data will be transferred from the clinical site in which it has been acquired (WCFT) to the UoL (Clinical Sciences Building, Room 2.23) for analysis on a secure, password protected networked University of Liverpool computer.

This data will include MRI, EEG and clinical/demographic data. All data will be anonymised from the point of extraction by staff at the clinical site and transferred to GA using a secure, encrypted external hard drive - at which point patients will sequentially be allocated study IDs as per the process outlined above.

All data in the study will be in a digital format as password protected computer files.

This will include data relating to acquired MRI or EEG information, demographic and clinical information collected both at baseline and follow up at the various time points. All of these files will be stored on a password protected University of Liverpool networked computer in pseudo-anonymised format from the point of acquisition using the naming format highlighted above.

Pseudo-anonymised digital data highlighted above will be archived on a secure, password protected networked University of Liverpool computer located on the second floor of the Clinical Sciences Building, Room 2.23 and will be stored for a period of 20 years following study completion. Following this time all data will be safely and appropriately

For peer review only - http://bmjopen.bmj.com/site/about/guidelines.xhtml

deleted. Data will be kept on a secure server that offers specialised storage of many terabytes of data per project which can comfortably accommodate all the data files generated from the study.

Data monitoring committee
As the nature of this study was observational, it was deemed appropriate that a data monitoring committee would not be required.

Informed Consent Process

All participants will be provided with a research information pack (appendix 1) describing the nature and goals of the research, and study consent form (appendix 2), which must be completed, signed and dated. We will not recruit participants who lack capacity to provide informed consent (e.g. those with intellectual disability or dementia). All participants will have the opportunity to discuss all aspects of the study with the Investigators. Participants will have as long as they require to consider their decision to volunteer for the research or not. The investigators contact details will be provided in the information pack. Participants are free to withdraw from the study at any time.

Confidentiality

The Chief Investigator will preserve the confidentiality of participants taking part in the study and is registered under the Data Protection Act.

Indemnity

The University of Liverpool holds Indemnity and insurance cover with Marsh UK LTD, which apply to this study.

Sponsor

The University of Liverpool will act as the main Sponsor for this study. Delegated responsibilities will be assigned to the NHS trusts taking part in this study.

Audits

The study may be subject to inspection and audit by the University of Liverpool under their remit as sponsor and other regulatory bodies to ensure adherence to GCP and the NHS Research Governance Framework for Health and Social Care (2nd edition).

Modification of the Protocol

Any modifications to the protocol which may impact on the conduct of the study, potential benefit of the patient or may affect patient safety, including changes of study objectives, study design, patient population, sample sizes, study procedures, or significant administrative aspects will require a formal amendment to the protocol. Such amendment will be agreed upon by research team and approved by the REC prior to implementation and notified to the health authorities in accordance with local regulations.

Dissemination

The aim will be to publish the results in high-quality peer-reviewed journals and to present at national and international conferences. We will target epilepsy-specific events (e.g. European Congress for Epileptology, International Epilepsy Congress, International League Against Epilepsy (ILAE) UK Chapter, American Epilepsy Society).

For each publication, only members of the research team who made a significant intellectual contribution to each piece of work will be considered as an author. This is in line with journal protocol. All authors share responsibility for the contents of the submitted manuscript.

Final data set

The CI and members of the research team will have access to the final cleaned data set that will be stored in accordance with secure data management methods as highlighted earlier in this protocol.

For peer review only

ACKNOWLEDGEMENTS

The authors are thankful to members of staff in the Institution of Translational Medicine (ITM), UoL for internal peer review of the original grant proposal and study costings for the fellowship application. The authors acknowledge all investigators at the planned recruitment site (WCFT) and Dr Kumar Das (Department of Neuroradiology, WCFT), Dr Shubhabrata Biswas (Department of Neuroradiology, WCFT), Dr Surjit Lyons-Nandra (Department of Neurophysiology, WCFT) for their support with the study.

We are incredibly grateful for the feedback of the patients and their families in designing this study as well as the support of all recruited participants without whom this study would not be possible.

FOOTNOTES

Author Contributions: GA contributed to the design of the MRI procedures and prepared the protocol for publication. LB and CTS contributed to the statistical elements of the study and sample size calculations. GA, TM and SSK contributed to the overall design of the study, leading on the setup of data collection methods at recruitment sites. GA, TM, SSK and MR contributed to the analysis plan. GA, TM and SSK conceived the study and led the development of the protocol. All authors provided critical intellectual input to the manuscript and have approved the final version for publication.

Funding: This work is supported by the Association of British Neurologists (ABN) and Guarantors of Brain (GoB) via a clinical research training fellowship awarded to GA and sponsored by the UoL (6107).

Disclaimer: The trial sponsor and funders had no role in trial design; collection, management, analysis and interpretation of data; writing of the report; and the decision to submit the report for publication, nor will they have ultimate authority over any of these activities. The views and opinions expressed herein are those of the authors and do not necessarily reflect those of the UoL, ABN, GoB and NHS.

Data sharing statement: Technical appendix, statistical code, and dataset will be made freely available from an appropriate data repository.

Competing interests: None declared.

Word Count: 3986 words.

For peer review only

REFERENCES

1. Hauser, W. A., & Beghi, E. (2008). First seizure definitions and worldwide incidence and mortality. *Epilepsia*, *49*, 8–12. https://doi.org/10.1111/j.1528-1167.2008.01443.x

2. Rizvi, S., Ladino, L. D., Hernandez-Ronquillo, L., & Téllez-Zenteno, J. F. (2017). Epidemiology of early stages of epilepsy: Risk of seizure recurrence after a first seizure. *Seizure*, *49*, 46–53. https://doi.org/10.1016/j.seizure.2017.02.006

3. Berg, A. T., & Shinnar, S. (1991). The risk of seizure recurrence following a first unprovoked seizure: a quantitative review. *Neurology*, *41*(7), 965–972. http://www.ncbi.nlm.nih.gov/pubmed/2067659

4. Debicki, D. B. (2017). Electroencephalography after a single unprovoked seizure. *Seizure*, *49*, 69–73. https://doi.org/10.1016/j.seizure.2017.03.001

5. Marson, A., Jacoby, A., Johnson, A., Kim, L., Gamble, C., Chadwick, D., & Medical Research Council MESS Study Group. (2005). Immediate versus deferred antiepileptic drug treatment for early epilepsy and single seizures: a randomised controlled trial. *The Lancet*, *365*(9476), 2007–2013. https://doi.org/10.1016/S0140-6736(05)66694-9

6. Bonnett, L. J., Tudur-Smith, C., Williamson, P. R., & Marson, A. G. (2010). Risk of recurrence after a first seizure and implications for driving: Further analysis of the multicentre study of early epilepsy and single seizures. *BMJ (Online)*, *341*(7785), 1260. https://doi.org/10.1136/bmj.c6477

7. Kim, L. G., Johnson, T. L., Marson, A. G., Chadwick, D. W., & MRC MESS Study group. (2006). Prediction of risk of seizure recurrence after a single seizure and early epilepsy: further results from the MESS trial. *The Lancet Neurology*, *5*(4), 317–322. https://doi.org/10.1016/S1474-4422(06)70383-0

8. Keller, S. S., Richardson, M. P., Schoene-Bake, J. C., O'Muircheartaigh, J., Elkommos, S., Kreilkamp, B., Goh, Y. Y., Marson, A. G., Elger, C., & Weber, B. (2015). Thalamotemporal alteration and postoperative seizures in temporal lobe epilepsy. *Annals of Neurology*, *77*(5), 760–774. https://doi.org/10.1002/ana.24376

9. Keller, S. S., Glenn, G. R., Weber, B., Kreilkamp, B. A. K., Jensen, J. H., Helpern, J. A., Wagner, J., Barker, G. J., Richardson, M. P., & Bonilha, L. (2017). Preoperative automated fibre quantification predicts postoperative seizure outcome in temporal lobe epilepsy. *Brain*, *140*(1), 68–82. https://doi.org/10.1093/brain/aww280

10. Walker, L. E., Frigerio, F., Ravizza, T., Ricci, E., Tse, K., Jenkins, R. E., Sills, G. J., Jorgensen, A., Porcu, L., Thippeswamy, T., Alapirtti, T., Peltola, J., Brodie, M. J., Park, B. K., Marson, A. G., Antoine, D. J., Vezzani, A., & Pirmohamed, M. (2017). Molecular isoforms of high-mobility group box 1 are mechanistic biomarkers for epilepsy. *The Journal of Clinical Investigation*, *127*(6), 2118–2132. https://doi.org/10.1172/JCI92001

11. Schmidt, H., Woldman, W., Goodfellow, M., Chowdhury, F. A., Koutroumanidis, M., Jewell, S., Richardson, M. P., & Terry, J. R. (2016). A computational biomarker of idiopathic generalized epilepsy from resting state EEG. *Epilepsia*, *57*(10), e200–e204. https://doi.org/10.1111/epi.13481

12. Peduzzi, P., Concato, J., Feinstein, A. R., & Holford, T. R. (1995). Importance of events per independent variable in proportional hazards regression analysis II. Accuracy and precision of regression estimates. *Journal of Clinical Epidemiology*, *48*(12), 1503–1510. https://doi.org/10.1016/0895-4356(95)00048-8

13. First Seizure Trial Group. (1993). Randomized clinical trial on the efficacy of antiepileptic drugs in reducing the risk of relapse after a first unprovoked tonic-clonic seizure. *Neurology*, *43*, 478–483. https://doi.org/10.1016/0736-4679(93)90655-Q

14. Browne R. H. (1995). On the use of a pilot sample for sample size determination. *Statistics in medicine*, *14*(17), 1933–1940. https://doi.org/10.1002/sim.4780141709

15.Sauerbrei, W. (1999). The Use of Resampling Methods to Simplify Regression Models in Medical Statistics. *Journal of the Royal Statistical Society: Series C (Applied Statistics)*, *48*(3), 313–329. https://doi.org/10.1111/1467-9876.00155

16. Keller SS, Highley JR, Garcia-Finana M, Sluming V, Rezaie R, Roberts N. Sulcal variability, stereological measurement and asymmetry of Broca's area on MR images. J Anat. 2007 Aug 29;211(4):534–55.

For peer review only - http://bmjopen.bmj.com/site/about/guidelines.xhtml

17. Keller SS, Gerdes JS, Mohammadi S, Kellinghaus C, Kugel H, Deppe K, et al. Volume Estimation of the Thalamus Using Freesurfer and Stereology: Consistency between Methods. Neuroinformatics. 2012 Oct 6;10(4):341–50.

18. Amann M, Andělová M, Pfister A, Mueller-Lenke N, Traud S, Reinhardt J, et al. Subcortical brain segmentation of two dimensional T1-weighted data sets with FMRIB's Integrated Registration and Segmentation Tool (FIRST). NeuroImage Clin. 2015;7:43–52.

19. O'Muircheartaigh, J., Vollmar, C., Traynor, C., Barker, G. J., Kumari, V., Symms, M. R., ... & Richardson, M. P. (2011). Clustering probabilistic tractograms using independent component analysis applied to the thalamus. *Neuroimage*, *54*(3), 2020-2032.Beckmann, C.F. and S.M. Smith, *Tensorial extensions of independent component analysis for multisubject FMRI analysis.* Neuroimage, 2005. 25(1): p. 294-311.

20. O'Muircheartaigh, J., Keller, S. S., Barker, G. J., & Richardson, M. P. (2015). White matter connectivity of the thalamus delineates the functional architecture of competing thalamocortical systems. *Cerebral Cortex*, *25*(11), 4477-4489.

21. Beckmann, C.F. and S.M. Smith, *Tensorial extensions of independent component analysis for multisubject FMRI analysis.* Neuroimage, 2005. 25(1): p. 294-311.

22. Glenn, G. R., Jensen, J. H., Helpern, J. A., Spampinato, M. V., Kuzniecky, R., Keller, S. S., & Bonilha, L. (2016). Epilepsy-related cytoarchitectonic abnormalities along white matter pathways. *J Neurol Neurosurg Psychiatry*, *87*(9), 930-936.

23. Kreilkamp, B. A., Weber, B., Richardson, M. P., & Keller, S. S. (2017). Automated tractography in patients with temporal lobe epilepsy using TRActs Constrained by UnderLying Anatomy (TRACULA). *NeuroImage: Clinical*, *14*, 67-76.

24. Bonilha, L., Helpern, J. A., Sainju, R., Nesland, T., Edwards, J. C., Glazier, S. S., & Tabesh, A. (2013). Presurgical connectome and postsurgical seizure control in temporal lobe epilepsy. *Neurology*, *81*(19), 1704-1710.

25. Bonilha, L., Jensen, J. H., Baker, N., Breedlove, J., Nesland, T., Lin, J. J., ... & Kuzniecky, R. I. (2015). The brain connectome as a personalized biomarker of seizure outcomes after temporal lobectomy. *Neurology*, *84*(18), 1846-1853.

26. Hutchings, F., Han, C. E., Keller, S. S., Weber, B., Taylor, P. N., & Kaiser, M. (2015). Predicting surgery targets in temporal lobe epilepsy through structural connectome based simulations. *PLoS computational biology*, *11*(12), e1004642.

27. Munsell, B. C., Wee, C. Y., Keller, S. S., Weber, B., Elger, C., da Silva, L. A., Nesland, T., Styner, M., Shen, D., & Bonilha, L. (2015). Evaluation of machine learning algorithms for treatment outcome prediction in patients with epilepsy based on structural connectome data. *NeuroImage*, *118*, 219–230. https://doi.org/10.1016/j.neuroimage.2015.06.008

28. Liu, M., Bernhardt, B. C., Hong, S. J., Caldairou, B., Bernasconi, A., & Bernasconi, N. (2016). The superficial white matter in temporal lobe epilepsy: a key link between structural and functional network disruptions. *Brain : a journal of neurology*, *139*(Pt 9), 2431–2440. https://doi.org/10.1093/brain/aww167

LIST OF FIGURES

1 Flow chart showing the recruitment process

APPENDICES

Appendix 1 – Participant information sheet

Appendix 2 – Participant consent form

For peer review only - http://bmjopen.bmj.com/site/about/guidelines.xhtml

For peer review only

For peer review only - http://bmjopen.bmj.com/site/about/guidelines.xhtml

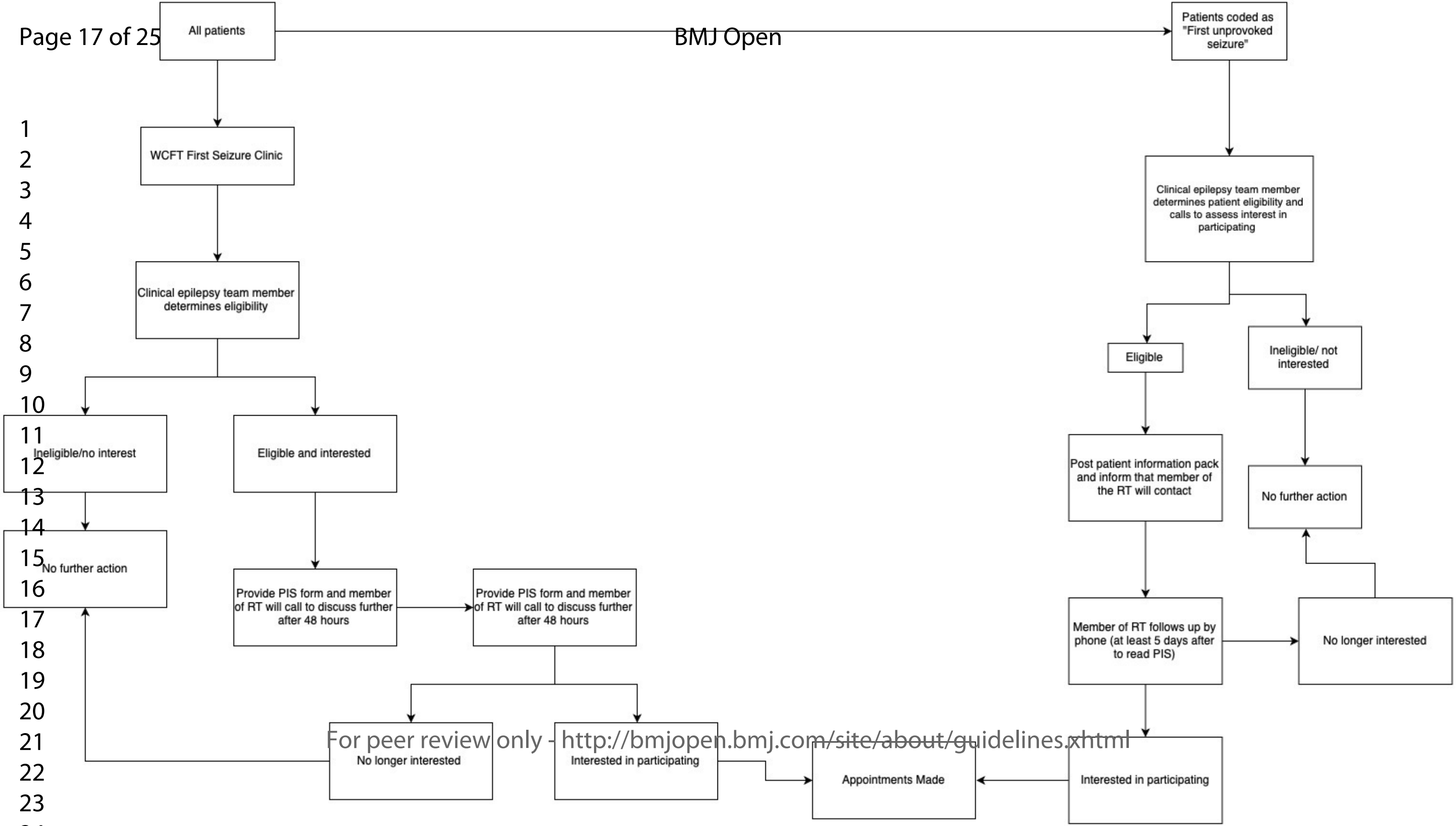

For peer review only - http://bmjopen.bmj.com/site/about/guidelines.xhtml

The Walton Centre NHS Foundation Trust
Lower Lane, Fazakerley
Liverpool, L9 7LJ, UK
Tel: 0151 525 3611
Fax: 0151 529 5500

Dr Guleed Adan
Clinical Research Fellow
2nd Floor, Neurological Science, Clinical Sciences Centre
Aintree University Hospital and The Walton Centre NHS Foundation Trusts
Lower Lane, Liverpool, L9 7LJ
Telephone: 0151 529 5943
Email: guleed@liverpool.ac.uk

PARTICIPANT INFORMATION SHEET FOR PATIENTS (PRAFUS-PIS-V4, 13/03/20)

Research study: Predicting recurrence after first unprovoked seizure (PRAFUS)

You are being invited to take part in a research study. Before you decide it is important for you to understand why the research is being done and what it will involve. Please take time to read the following information carefully. Talk to others about the study if you wish.

- Part 1 tells you the purpose of this study and what will happen to you if you take part.
- Part 2 gives you more detailed information about the conduct of the study.

Ask us if there is anything that is not clear or if you would like more information. Take time to decide whether you wish to take part.

PART 1

What is the purpose of the study?

People who have had a first unprovoked seizure may have an EEG and an MRI scan performed. At the moment, we find that many people with a first seizure often have a normal MRI and EEG. We think this is because we do not yet know the best way to carry out EEG and MRI scanning for people with first unprovoked seizure, and that the current use of scans may not be detailed enough. We have developed some new EEG and MRI scanning methods, and would like to try them out in people who have had a first unprovoked seizure. We hope that these new scans will provide us with a more detailed picture of the structure and function of the brain, which may be very important for people who have a first seizure so that we can better predict those that will go on to have further seizures and therefore develop epilepsy. We are hoping that our EEG and MRI scans can provide information on why some people have further seizures and others do not. We will also be taking blood and saliva samples that will be analysed for novel markers of inflammation that may help to identify whether increased levels of inflammation in the body is linked to the chance of having future seizures.

Why have I been chosen?

You have been chosen because we know you have been recently diagnosed with a first unprovoked seizure.

Do I have to take part?

No. It is entirely up to you to decide whether or not to take part. If you do, you will be given this information sheet to keep and be asked to sign a consent form. You are still free to withdraw at any time and without giving a reason. A decision to withdraw at any time, or a decision not to take part, will not affect the standard of clinical care you receive. If you express an interest in participating in this research, a member of the research team will contact you by your preferred method of correspondence.

What will happen to me if I take part?

We will send you an appointment for the following:

For peer review only - http://bmjopen.bmj.com/site/about/guidelines.xhtml

The Walton Centre NHS Foundation Trust
Lower Lane, Fazakerley
Liverpool, L9 7LJ, UK
Tel: 0151 525 3611
Fax: 0151 529 5500

(1) An MRI brain scan at LiMRIC or the Walton Centre. The MRI scan will last about 30 minutes and will involve you lying still and relaxing in the scanner. If your scan takes place at the LiMRIC, you will have some advanced brain scans performed that will involve some extra time to the standard clinical scans that are used currently. This will not compromise your clinical care and your scan will still be analysed and reported by a neuro-radiology consultant at the Walton Centre. Your report will still be returned to the neurology consultant that you are under the care of. A summary of the details of your MRI scan will be sent to you and to your GP as per the Walton Centre's standard clinical practice.

(2) After or before your MRI scan we will take a maximum of three blood tubes (containing nine millilitres each), which is the equivalent of an eggcup full of blood in total. We will also take a small salivary swab from the inside of the cheek. This will take around five minutes. Blood and saliva samples will be analysed and stored in a biobank (freezer) at the LiMRIC for a period of five years or until all of the sample has been used up, whichever is sooner.

(3) Finally, an EEG scan at the Walton Centre, where you will have already been seen by a specialist. This will last around one hour and will involve electrodes being placed on the surface of your head. You will be seated upright, awake and will be relaxed while measurements are taken. The EEG test will likely be on a different day to the MRI scan and blood sample.

There are no more scans or tests after this. A clinical member of the research team will contact you at four different time points after your first seizure: 6 months, 12 months, 18 months and 24 months. This will involve asking basic and brief questions about any further seizures you may have had.

What do I have to do?
All you need to do is sit and relax for the EEG scan and relax and lie still in the MRI scanner. A qualified health care professional will take a sample of your blood and saliva swab before or after your MRI scan.

What are the other possible disadvantages and risks of taking part?
The technique of MRI has been in use in medicine for about 30 years and has shown to be safe. It does not involve any radiation. In some people, there are times when it is not safe to be scanned. For example, in the first three months of pregnancy, or when there are surgical clips inside the brain, or if there is a heart pacemaker or Vagus Nerve Stimulator fitted. Furthermore, the scanner may get warm and noisy, and may not be suitable for sufferers of claustrophobia. However, rest assured, we will discuss with you thoroughly prior to the scan to identify any reasons why you should not have the scan. We will also thoroughly review with you on the day of scanning whether there is any possible risk for you. Earplugs are used to reduce the impact of scanner noise.

What are the possible benefits of taking part?
There may be no direct benefit to you from taking part. We hope that we will gain useful information predicting further seizures in people like yourself from these new scanning techniques, but we cannot be certain about this.

What if there is a problem?
Any complaint about the way you have been dealt with during the visit will be addressed. The detailed information on this is given in Part 2.

Will my taking part in the study be kept confidential?
Yes. All the information about your participation in this study will be kept confidential. The details are included in Part 2.

Contact Details:

For peer review only

The Walton Centre NHS
NHS Foundation Trust

The Walton Centre NHS Foundation Trust
Lower Lane, Fazakerley
Liverpool, L9 7LJ, UK
Tel: 0151 525 3611
Fax: 0151 529 5500

For further information, please contact any of the team running this research project:

Dr Guleed Adan
Clinical Research Fellow
2nd Floor, Neurological Science, Clinical Sciences Centre
Aintree University Hospital and The Walton Centre NHS Foundation Trusts
Lower Lane, Liverpool, L9 7LJ
Telephone: 0151 529 5943
Email: guleed@liverpool.ac.uk

Dr. Simon Keller
Senior Lecturer and Researcher in Neuroimaging
2nd Floor, Neurological Science
Clinical Sciences Centre
Aintree University Hospital and The Walton Centre
NHS Foundation Trusts
Lower Lane, Liverpool, L9 7LJ
Telephone: 07795617348
Email: simon.keller@liv.ac.uk

Professor Anthony Marson
Professor of Neurology
2nd Floor, Neurological Science
Clinical Sciences Centre
Aintree University Hospital and The Walton Centre
NHS Foundation Trusts
Lower Lane, Liverpool, L9 7LJ
Telephone: 0151 529 5770
Email: a.g.marson@liv.ac.uk

This completes Part 1 of the Information Sheet.
If the information in Part 1 has interested you and you are considering participation, please continue to read the additional information in Part 2 before making any decision.

For peer review only

The Walton Centre NHS
NHS Foundation Trust

The Walton Centre NHS Foundation Trust
Lower Lane, Fazakerley
Liverpool, L9 7LJ, UK
Tel: 0151 525 3611
Fax: 0151 529 5500

PART 2

What will happen if I don't want to carry on with the study?
You can withdraw from this research study at any time, even while you are having the tests done. This will not affect your treatment in any way. Even if you withdraw from the study, we would still like to use any information we might already have collected.

What if new information becomes available?
It is unlikely that any new information will become available while you are taking part in the study. Because we will be taking pictures of your brain with the MRI scan, occasionally we will have unexpected findings that none of us suspected. The pictures are reviewed by experienced doctors, called neuroradiologists who specialise in looking at pictures of brain. If there are any unexpected findings that need further tests, he/she will write to your GP or specialist. The doctor will then contact you if further tests are required. However, unexpected findings on MRI scans are rare.

What if there is a problem?
If you have a concern about any aspect of this study, you should ask to speak with the researchers who will do their best to answer your questions. If you remain unhappy and wish to complain formally, you can do this through the NHS Complaints Procedure. Details can be obtained from The Walton Centre NHS Foundation Trust:

Patient Experience Team
Sid Watkins Building
The Walton Centre
Lower Lane, Fazakerley, Liverpool, L9 7LJ
Tel: 0151 556 3090/3091
Email: patientexperienceteam@thewaltoncentre.nhs.uk

Further information on official complaints can be found here: https://www.thewaltoncentre.nhs.uk/362/comments-complaints-and-compliments.html

If something does go wrong and you are harmed during the research study there are no special compensation arrangements. If you are harmed and this is due to someone's negligence then you may have grounds for a legal action for compensation against the hospital, but you may have to pay your legal costs. The normal National Health Service complaints mechanisms will still be available to you.

Can I speak to anyone else?
If you would like to speak to someone not part of the research team about MRI scanning in general, please contact:

Professor Graham Kemp
Director, The Liverpool Magnetic Resonance Imaging Centre (LiMRIC)
Pembroke Place, Liverpool, L69 3GE
Telephone: 0151 794 5635; Email: gkemp@liverpool.ac.uk

The Walton Centre NHS Foundation Trust
Lower Lane, Fazakerley
Liverpool, L9 7LJ, UK
Tel: 0151 525 3611
Fax: 0151 529 5500

Who is organising and funding the research?
This research is being organised Dr Guleed Adan and is sponsored by The University of Liverpool (see below). A Clinical Research Training Fellowship awarded to Dr Adan, supervised by Prof. Marson and Dr. Keller is funding this research. Neither the research team nor your doctor receives any payment if you take part.

Will my taking part in this study be kept confidential?
Yes. We will collect information about you that could identify you personally (for example, because the information includes your name or date of birth). We will also collect information about you because we believe it might be relevant to understanding the research (e.g. information about your epilepsy, medicine, any previous scans and EEGs, dates and times of seizures near to the MRI scan). This information will be stored in "pseudo-anonymised" form (which means that your name, address and other personal details will be linked with the information we use in the research, but will not be directly accessible during the research) on computers owned by the hospital and on computers owned by the University of Liverpool. These computers will be securely controlled by the research team under the direct responsibility of Dr. Simon Keller and Professor Tony Marson, and no-one outside the team will have access to your information. We will use the information we collect to answer the questions relevant to this research project. Blood and saliva samples will be stored in anonymous form in dedicated research laboratories at the University of Liverpool until samples are used up.

The data that we collect from you (MRI scans, EEG scans, blood and saliva samples) will be kept for future research. All of this data will not include any identifiable information about you. The data may be made available to other researchers that work with the study team, but at no point is any information about who you are indicated or shared.

In the future, it is possible we might have new research questions that could be answered by looking at your information in new ways. We would seek approval from the Research Ethics Committee to use your information for new research projects. If the Research Ethics Committee believed we should contact you again to ask your permission to re-use your information, we will do so. The hospital has a duty to ensure research conducted here is of a high standard and auditors from the hospital may need to review any information we hold about you. The auditors will maintain the highest standards of confidentiality. Procedures for handling, processing, storage and destruction of your data are compliant with the Data Protection Act 1998.

General Data Protection Regulation
The University of Liverpool is the sponsor for this study based in the United Kingdom. We will be using information from you and your medical records in order to undertake this study and will act as the data controller for this study. This means that we are responsible for looking after your information and using it properly. The University of Liverpool will keep identifiable information about you for 20 years after the study has finished if you consent to be contacted about future studies. If you do not consent to being contacted about future relevant studies, your identifiable information will not be kept beyond 5 years after the study has finished.

Your rights to access, change or move your information are limited, as we need to manage your information in specific ways in order for the research to be reliable and accurate. If you withdraw from the study, we will keep the information about you that we have already obtained. To safeguard your rights, we will use the minimum personally-identifiable information possible.

You can find out more about how we use your information by contacting Dr Guleed Adan (or alternative team member). Our Data Protection Officer is Victoria Heath and you can contact them at V.Heath@liverpool.ac.uk.

The University of Liverpool will use your name, NHS number and contact details to contact you about the research study, and make sure that relevant information about the study is recorded for your care, and to

The Walton Centre NHS Foundation Trust
Lower Lane, Fazakerley
Liverpool, L9 7LJ, UK
Tel: 0151 525 3611
Fax: 0151 529 5500

oversee the quality of the study. Individuals from the University of Liverpool and regulatory organisations may look at your medical and research records to check the accuracy of the research study. The Walton Centre will pass these details to the University of Liverpool along with the information collected from you and your medical records. The only people in The University of Liverpool who will have access to information that identifies you will be people who need to contact you to arrange an appointment for the research study investigations or audit the data collection process. The people who analyse the information will not be able to identify you and will not be able to find out your name, NHS number or contact details.

When you agree to take part in a research study, the information about your health and care may be provided to researchers running other research studies in this organisation and in other organisations. These organisations may be universities, NHS organisations or companies involved in health and care research in this country or abroad. Your information will only be used by organisations and researchers to conduct research in accordance with the UK Policy Framework for Health and Social Care Research (https://www.hra.nhs.uk/planning-and-improving-research/policies-standards-legislation/uk-policy-framework-health-social-care-research/).

This information will not identify you and will not be combined with other information in a way that could identify you. The information will only be used for the purpose of health and care research and cannot be used to contact you or to affect your care. It will not be used to make decisions about future services available to you, such as insurance.

Involvement of your doctor
The doctor looking after you in the hospital will be aware of your participation in this research study.

What will happen to the results of the research study?
The scientific results of this research study will be published in scientific and medical journals and may be discussed at scientific meetings. You will not be personally identified in any way.

Who has reviewed the study?
North East - Tyne and Wear South Research Ethics Committee has reviewed this study and given a favourable ethical opinion for this research.

You will be given a copy of the information sheet and a copy of your signed consent form to keep.

Thank you for considering taking part in this research project and thank you for taking the time to read the information sheets.

For peer review only

The Walton Centre NHS
NHS Foundation Trust

The Walton Centre NHS Foundation Trust
Lower Lane, Fazakerley
Liverpool, L9 7LJ, UK
Tel: 0151 525 3611
Fax: 0151 529 5500

CONSENT FORM

Short title of Project: Predicting recurrence after first unprovoked seizure (PRAFUS)
Version 2=3: PRAFUS-CON-V3; 13/03/2020
IRAS Project ID: 279362**;** Research ethics committee ID:

Name of Chief Investigator: Prof. Tony Marson

	**Please initial box**
1. I confirm that I have read and understand the participant information sheet dated 13/03/20 (Version 4: **PRAFUS-PIS-V4**) for the above study. I have had the opportunity to consider the information, ask questions and have had these answered satisfactorily.	☐
2. I understand that my participation is voluntary and that I am free to withdraw at any time, without giving any reason, without my medical care or legal rights being affected.	☐
3. I understand that relevant sections of any of my medical notes and data collected during the study may be looked at by responsible individuals from regulatory authorities or from the NHS Trust, where it is relevant to my taking part in this research. I give permission for these individuals to have access to my records.	☐
4. I agree for a blood and saliva sample to be taken and used for future research. I understand that blood and saliva samples will be stored in anonymous form in dedicated research laboratories at the University of Liverpool for a period of five years or until samples are depleted, whichever is sooner.	☐
5. I agree for the research team to share anonymised data collected in this study with other researchers; I understand that I will not be identified in anyway.	☐
6. I agree to be contacted by a clinical member of the research team at 6, 12, 18 and 24 months after my MRI scan by telephone who will ask me brief questions about my seizures and medication.	☐
7. I agree that I may be allocated to have advanced MRI brain scans to be performed at the University of Liverpool, which will include some additional scanning (sequences) to the standard clinical scans that would have otherwise been performed at the Walton Centre. I understand that my clinical care will not be compromised should I agree to this.	☐

When completed, 1 for patient; 1 for researcher site file; 1 (original) to be kept in medical notes

For peer review only

The Walton Centre NHS
NHS Foundation Trust

The Walton Centre NHS Foundation Trust
Lower Lane, Fazakerley
Liverpool, L9 7LJ, UK
Tel: 0151 525 3611
Fax: 0151 529 5500

8. I agree to be contacted in the future should another scanning study become available. ☐

Preferred method of contact:

Address	Email
Home telephone	Mobile telephone

9. I agree to take part in the above study. ☐

Name of Participant	Date	Signature
Name of legal guardian or parent<br>***(if participant is under 18)***	Date	Signature
Name of Person taking consent<br>(if different from researcher)	Date	Signature
Researcher	Date	Signature

When completed, 1 for patient; 1 for researcher site file; 1 (original) to be kept in medical notes

For peer review only

BMJ Open

A protocol for an observational cohort study investigating biomarkers predicting seizure recurrence following a first unprovoked seizure in adults.

Journal:	*BMJ Open*
Manuscript ID	bmjopen-2022-065390.R1
Article Type:	Protocol
Date Submitted by the Author:	08-Nov-2022
Complete List of Authors:	Adan, Guleed; University of Liverpool, Department of Pharmacology and Therapeutics; The Walton Centre NHS Foundation Trust<br>de Bézenac, Christophe; University of Liverpool, Molecular and Clinical Pharmacology<br>Bonnett, Laura; University of Liverpool Department of Biostatistics<br>Pridgeon, Michael; The Walton Centre NHS Foundation Trust<br>Biswas, Shubhabrata; The Walton Centre NHS Foundation Trust<br>Das, Kumar; The Walton Centre NHS Foundation Trust<br>Richardson, Mark P.; King's College London Institute of Psychiatry Psychology and Neuroscience, Department of Basic and Clinical Neuroscience<br>Laiou, Petroula; King's College London Institute of Psychiatry Psychology and Neuroscience, Department of Basic and Clinical Neuroscience<br>Keller, Simon S.; University of Liverpool, Department of Pharmacology and Therapeutics; The Walton Centre NHS Foundation Trust<br>Marson, Tony; University of Liverpool, Department of Pharmacology and Therapeutics; The Walton Centre NHS Foundation Trust
**Primary Subject Heading**:	Neurology
Secondary Subject Heading:	Epidemiology, Neurology
Keywords:	Epilepsy < NEUROLOGY, NEUROPHYSIOLOGY, Magnetic resonance imaging < RADIOLOGY & IMAGING

SCHOLARONE™
Manuscripts

A protocol for an observational cohort study investigating biomarkers predicting seizure recurrence following a first unprovoked seizure in adults.

Guleed Adan[1,2,],Christophe de Bézenac[1,2],Laura Bonnett[3],Michael Pridgeon[2], Shubhabrata Biswas[2], Kumar Das[2], Mark P. Richardson[4], Petroula Laiou[4],Simon S. Keller[1,2] Tony Marson[1,2]

1. Department of Pharmacology and Therapeutics, Institute of Systems, Molecular and Integrative Biology, University of Liverpool.

2. The Walton Centre NHS Foundation Trust, Lower Lane, Liverpool, L9 7LJ, United Kingdom.

3. Department of Biostatistics, University of Liverpool, Liverpool, L69 3BX, United Kingdom.

4. Department of Basic and Clinical Neuroscience, Institute of Psychiatry, Psychology and Neuroscience, King's College London, London, UK

Trial registration:

NIHR Clinical Research Network's (CRN) Central Portfolio Management System (CPMS) - 44976

World Health Organization Trial Registration Data Set:

Data category	Information
Primary registry and trial identifying number	NIHR CRN CPMS 44976
Date of registration in primary registry	11th March, 2020
Secondary identifying numbers	IRAS 279362, UoL0015106107, REC reference 20/NE/0078
Source(s) of monetary or material support	Association of British Neurologists and Guarantors of Brain
Primary sponsor	Prof Neil French, University of Liverpool [sponsor@liv.ac.uk]
Contact for public queries	Dr Guleed Adan [guleed@liv.ac.uk]
Contact for scientific queries	Dr Guleed Adan [guleed@liv.ac.uk]<br>Department of Pharmacology and Therapeutics, University of Liverpool
Public title	Predicting recurrence after a first unprovoked seizure (PRAFUS)
Scientific title	Predicting recurrence after a first unprovoked seizure — a prospective observational cohort study
Countries of recruitment	England, UK
Health condition(s) or problem(s) studied	First unprovoked seizure, epilepsy, seizure recurrence
Intervention(s)	None.
Key inclusion and exclusion criteria	Ages eligible for study: ≥16 years<br>Sexes eligible for study: both<br>Accepts healthy volunteers: no

For peer review only

Data category	Information
	Inclusion criteria: adult patient (≥ 18 years), diagnosed with a first unprovoked seizure of any semiology, maximum of 8 weeks since first seizure
	Exclusion criteria: provoked seizures, dissociative seizures, progressive or inflammatory neurological disorder, previous neurosurgery
Study type	Observational
	Primary purpose: development of a predictive prognostic model
Date of first enrolment	17th August 2020.
Target sample size	100
Primary outcome(s)	Time to seizure recurrence (follow up time: 24 months)

Date and protocol version identifier:

Version 1: 23/09/2019, University of Liverpool.
Version 2: 02/10/2019, University of Liverpool.
Version 3: 31/10/2019, University of Liverpool.
Version 4: 13/11/2019, University of Liverpool.
Version 5: 05/12/2019, University of Liverpool.
Version 6: 17/01/2020, University of Liverpool.
Version 7: 09/03/2020, University of Liverpool.
Version 8: 13/03/2020, University of Liverpool.
Version 9: 29/05/2020, University of Liverpool.

Correspondence to:

Dr. Guleed Adan
University of Liverpool
Dept of Neurological Sciences
Clinical Sciences Centre
Lower Lane
Liverpool
L9 7LJ
UK
Guleed.adan@liverpool.ac.uk
Tel: +44 (0)7783844562

For peer review only - http://bmjopen.bmj.com/site/about/guidelines.xhtml

ABSTRACT

Introduction:

A first unprovoked seizure is a common presentation, reliably identifying those that will have recurrent seizures is a challenge. This study will be the first to explore the combined utility of serum biomarkers, quantitative EEG and quantitative MRI to predict seizure recurrence. This will inform patient stratification for counselling and the inclusion of high-risk patients in clinical trials of disease modifying agents in early epilepsy.

Methods and analysis: 100 patients with first unprovoked seizure will be recruited from a tertiary neuroscience centre and baseline assessments will include structural MRI, EEG, and a blood sample. As part of a nested pilot study, a subset of 40 patients will have advanced MRI sequences performed that are usually reserved for patients with refractory chronic epilepsy. The remaining 60 patients will have standard clinical MRI sequences. Patients will be followed up every 6 months for a 24-month period to assess seizure recurrence. Connectivity and network-based analyses of EEG and MRI data will be carried out and examined in relation to seizure recurrence. Patient outcomes will also be investigated with respect to analysis of high mobility group box-1 (HMGB1) from blood serum samples.

Ethics and dissemination: This study was approved by North East – Tyne & Wear South Research Ethics Committee (20/NE/0078) and funded by an Association of British Neurologists (ABN) and Guarantors of Brain (GoB) clinical research training fellowship. Findings will be presented at national and international meetings published in peer-reviewed journals.

Keywords: First unprovoked seizure, Prognostic modelling, Brain connectivity, Quantitative MRI, Biomarker

Strengths and Limitations

- This will be the first study to prospectively investigate how brain structural and physiological architecture and connectivity in adults influences seizure recurrence following a first unprovoked seizure.
- The study is expected to provide insights into the biology underlying epileptogenesis, and to lead to the development of prognostic markers of seizure recurrence following a first unprovoked seizure.
- Expected recruitment has been based on records of past diagnosis and while the study is expected to recruit well, unexpected under-recruitment is possible and would be a barrier to timely completion.
- A second potential limitation of this study is the potential for participant attrition and loss of patient follow up at multiple points over 24 months; missing data could impact on the validity of study conclusions.

For peer review only

For peer review only - http://bmjopen.bmj.com/site/about/guidelines.xhtml

INTRODUCTION

Background and rationale

A first unprovoked seizure is a common presentation; the annual incidence is between 50 and 70 per 100,000[1]. At least 10% of the population will have at least one seizure[2] and approximately 50% will have a recurrence[3]. A major challenge is to reliably identify those that will have recurring seizures, to better inform treatment decisions and counselling[4]. This will be increasingly important as we try to develop disease-modifying treatments. One paradigm will be to test new and repurposed treatments early in the disease course, using a design similar to the Multi-centre Study of Early Epilepsy and Single Seizures (MESS)[5]. Efficient trial designs will require the recruitment of people at high risk of seizure recurrence and this study will aid the development of EEG, MRI and blood biomarkers to do so.

Various investigations are used in the diagnostic work up of patients with first unprovoked seizures including electroencephalogram (EEG) and neuroimaging, which have been shown to have some prognostic value, but current prognostic models lack precision to reliably stratify patients. Prognostic models of data from the Multi-centre Study of Early Epilepsy and Single Seizures (MESS) study[6,7] identified epileptiform EEG abnormality, neurological deficit and abnormal magnetic resonance imaging (MRI) as significant prognostic factors. For these models, EEG and MRI were simply classified as normal, abnormal or 'non-specific'. It is now possible to use advanced quantitative approaches to analyse EEG and MRI and use them as continuous measures of neurophysiological function and anatomical variation, as proposed in this study.

In this study we will investigate emerging epilepsy biomarkers in first seizure populations. We already know that patients with an MRI lesion have a higher risk of recurrence[6], however, advanced quantitative MRI analysis has never been used in this population. These methods have been demonstrated by the Liverpool Epilepsy Research Group to be effective in predicting seizure outcomes in pre-surgical patients with temporal lobe epilepsy[8,9]. High-mobility group box 1 (HMGB1) is a key neuro-inflammatory mediator in epilepsy. Increased levels of expression of HGMB1 have been shown by the Liverpool group to be associated with increased seizure frequency in newly diagnosed epilepsy[10]. Computational analysis of resting-state EEG has been shown to reliably differentiate between cases of idiopathic generalised epilepsy and healthy controls[11].

Trial design

A prospective, observational cohort study that includes a nested exploratory study;

1. a prospective study in which those EEG and MRI biomarkers will be further refined and validated. Inflammatory biomarkers in the blood and saliva will also be assessed.
2. a nested exploratory study in which the utility of advanced quantitative MRI biomarkers in patients with a first unprovoked seizure will be assessed.

Study setting

Participants will be recruited from a tertiary academic neuroscience centre in England.

Rationale for study

Hypothesis

Following a first unprovoked seizure, patients at high risk of a recurrence can be identified using a combination of EEG, MRI, blood serum inflammatory biomarkers and clinical factors.

Overall aims

The overall aim is to undertake a multi-modal investigation of brain structure, connectivity and inflammation in adults with a first unprovoked seizure. The proposed project will provide new insights into the biology underlying first unprovoked seizures in humans whilst also allowing us to develop prognostic markers of seizure recurrence. This research will take place in the context of collaboration between researchers with an internationally respected reputation for research in epilepsy, and in an environment with demonstrated excellence in recruitment of patients with both first seizure and newly diagnosed epilepsy into research studies and clinical trials.

Prospective sub-study

For peer review only

Objectives:

1. To externally validate the prognostic markers of seizure recurrence following first unprovoked seizure that have been developed in a retrospective study
2. To identify blood and salivary biomarkers of seizure recurrence after first unprovoked seizure
3. To identify, in a subset of 40 patients, potential biomarkers of seizure recurrence from advanced quantitative image analysis*

NB: The methods described in this protocol apply to both the prospective study and the exploratory study unless otherwise stated.

METHODS AND ANALYSIS

Outcomes

The primary outcome event being studied is seizure recurrence following a first unprovoked seizure. We propose to use a multivariable prognostic model which evaluates the utility of multi-modal biomarkers of seizure recurrence using a time-to-event-outcome, with the event of interest being seizure recurrence. Seizure recurrence will be identified at follow-up intervals of 6, 12, 18 and 24 months.

Population

100 patients with a first unprovoked seizure will be recruited from "first seizure clinics" at the WCFT. Suitable patients will be identified by treating clinicians directly from clinic and also from the electronic patient records. A summary of the recruitment process is shown in Figure 1 and highlighted in more detail in the following section. Only patients that satisfy the inclusion and exclusion criteria below will be recruited into the study.

(Insert Figure 1 here)

Inclusion criteria:

- Aged over 16 years*
- Diagnosis of a first unprovoked seizure (of any semiology or type, including status epilepticus) made at WCFT clinic by a member of the clinical epilepsy team.
- Maximum of eight weeks since first unprovoked seizure

**recruited participants aged between 16-18 years will have appropriate parental consent sought in addition to their own, additional space for appropriate parental signatures will be available on the standard consent form used in the study to allow for this eventuality.*

Exclusion criteria:

- Provoked seizures (e.g. alcohol or drug induced)
- Non-epileptic seizures
- Acute symptomatic seizures (e.g. acute brain haemorrhage or brain injury)
- Known progressive neurological disease (e.g. brain tumour, Alzheimer's disease).
- Known inflammatory neurological condition (specifically multiple sclerosis or sarcoidosis)
- Previous neurosurgery

For peer review only - http://bmjopen.bmj.com/site/about/guidelines.xhtml

- None ambulatory patients with known significant issues with mobility which impairs ability to independently transfer onto MRI scanner e.g. hoist transfer dependent
- Significant medical co-morbidity e.g. pre-existing severe cardiovascular or respiratory disease that would make them unsuitable for the prolonged supine positioning required for MRI scanning
- Inability to understand written or spoken English

Withdrawal criteria:

- Participants may withdraw their participation in this study by contacting the CI or a member of the study research team at any time.

Recruitment via the outpatient/telemedicine clinic:

A clinical member of the wider research team (i.e. consultant neurologist, epilepsy nurse or neurology specialist trainee) will enquire whether eligible patients would be interested in participating in this study at the time of consultation during outpatient/telemedicine clinics.

Recruitment via the electronic patient record system:

Patients who have been coded as having a first unprovoked seizure from the WCFT electronic patient records will be assessed according to the eligibility criteria mentioned previously. Suitable patients will then be contacted by telephone by a clinical member of the epilepsy team at the Walton Centre to enquire whether eligible patients would be interested in participating in this study.

Procedures/Assessments

Summary of the procedures for each participant (Table 1):

Table 1. Summary of the procedures for each participant:

Procedure	Location	Duration	Number of examinations
1.MRI	*LiMRIC, UoL or WCFT	1 hour, including safety examination and set up	1
2.Blood extraction and saliva collection	*LiMRIC, UoL or WCFT	5 minutes	1
3.EEG	Neurophysiology, WCFT	1 hour, including set up	1
4.Telephone questionnaire	Home	5 minutes	4 (6, 12, 18 and 24 months after index event)

**Patients in the exploratory study within the prospective cohort will have their imaging, saliva and blood sampling performed at the LiMRIC, UoL only instead of the WCFT.*

Study timeline

For peer review only - http://bmjopen.bmj.com/site/about/guidelines.xhtml

The proposed study will last three years and will be split into three phases which are outlined below. The study started August 2020 with a planned end date of August 2023. We require a recruitment period long enough to recruit a sufficient number of patients with first unprovoked seizure and a follow up period long enough to establish likely seizure recurrence.

Phase 1 (Ph1; *month 1-3*) is an initial *3-month* period dedicated to project set-up and optimisation of the MRI protocol. MRI optimisation will include technical development MRI scanning of phantoms and human volunteers to ensure the MRI sequences are adequate for the study. Necessary sponsorship and ethical approval will be sought during phase 1.

Phase 2 (Ph2; *month 4-24*) is a *20-month* period that includes participant recruitment, baseline clinical data collection, MRI, EEG, blood and saliva acquisition for all recruited participants.

Phase 3 (Ph3; *month 10-34)* is a 24-month patient follow-up period during which time all seizure recurrence information will be recorded by telephone by at 6,12,18 and 24 months after the index event.

Sample size calculation

We propose to use a multivariable prognostic model using time-to-event-outcome with the event of interest being seizure recurrence. With multiple variables of equal interest, development of a 'standard' sample size formulae is problematic. Therefore, we propose to use an events per variable calculation. The most often cited recommendation is the rule of '10 events per variable (EPV)'[12]. We will recruit 100 patients therefore, with an assumed 2 year seizure recurrence rate of 51%[13], we would be able to include up to 5 predictor levels.

40 of the 100 patients recruited will have additional advanced MRI sequences performed as part of the nested exploratory study. A sample size of 40 was chosen largely to cost limitations and given that it would satisfy the sample size flat rule of having at least 30 participants[14]. For convenience, the first 40 participants recruited will be receiving advanced MRI sequences.

Data acquisition*

In total, we will prospectively perform clinical MRI scans, routine EEG, saliva and blood sample investigations in all of the 100 patients recruited prospectively. A table detailing data collection at the various timepoints of the study is presented below (Table 2).

Table 2. Study Timepoints

		Follow Up Schedule			
Procedures	Baseline (T0)[1]	T0+6 months	T0+12 months	T0+18 months	T0+24months
Signed Consent Form	X				
Assessment of Eligibility Criteria	X				
Contact details	X				
Review of Medical History and demographics including:<br>• Age	X				

For peer review only - http://bmjopen.bmj.com/site/about/guidelines.xhtml

		Follow Up Schedule			
Procedures	Baseline (T0)[1]	T0+6 months	T0+12 months	T0+18 months	T0+24months
• Gender<br>• Seizure type<br>• Neurological deficit<br>• Febrile seizures<br>• Family history of epilepsy					
Investigations (EEG, MRI, blood and saliva sampling)	X				
Review of seizure occurrence by telephone		X	X	X	X

*In addition to the standard clinical MRI sequences which all 100 participants will have performed, advanced quantitative MRI scanning will be performed at the LiMRIC main campus for 40 patients that are part of the cohort in the exploratory sub-study outlined previously.

All EEG data collection will take place at the WCFT, in the department of neurophysiology.

MRI

All 100 participants recruited will have clinical MRI scans performed which will include the following sequences:

1. Conventional 2D T2-weighted fast spin echo and fast Fluid Attenuated Inversion Recovery scans, for incidental findings screening, and detection of gross pathology (together with localizer ~10 minutes)

2. 3D T1-weighted MPRAGE scan with isotropic voxel size of 1 mm x 1 mm x 1mm (~10 minutes);

As part of the exploratory nested study looking at the predictive utility of advanced imaging in first seizure patients, 40 patients will have advanced MRI scans performed (max 30 minutes) and will consist of:

1. Conventional 2D T2-weighted fast spin echo and fast Fluid Attenuated Inversion Recovery scans, for incidental findings screening, and detection of gross pathology (together with localizer ~10 minutes)

2. 3D T1-weighted MPRAGE scan with isotropic voxel size of 1 mm x 1 mm x 1mm (~10 minutes);

3. high resolution diffusion kurtosis imaging (DKI) sequence with at least 60 isotropically distributed gradient directions, three b values (b=0, 1000 and 2000) and maximum voxel size of 2 mm x 2 mm x 2mm (~10 minutes).

MRI Safety Criteria

All participants who are having an MRI scan as part of the study will have a completed MRI safety screening as a pre-requisite.

For peer review only - http://bmjopen.bmj.com/site/about/guidelines.xhtml

All participants will be examined by a radiographer and will complete a safety checklist that is designed to identify whether a participant has internal bodily metal, which could pose a hazard during MRI scanning. All removable bodily metal will be removed before scanning.

Blood extraction

All 100 patients will have blood collected for analysis in a Lithium-Heparin bottles or serum separator tubes (9mls). A maximum of 27 milliliters of blood (3 x 9ml vials) will be obtained from each participant. Samples will be obtained by a healthcare professional trained in phlebotomy. A standard operating procedure for blood sampling including aseptic technique will be utilised by all practitioners involved in the study. Blood samples will be centrifuged within 15 minutes of collection or stored overnight at 4°c for centrifuge the following day. 250 µl aliquots will then be transferred to appropriate tubes and stored at approximately -80 degrees Celsius prior to bioanalysis. This process is identical to other studies in the group running in parallel with REC approval (REC reference 17/NW/0342 and 19/NW/0384).

EEG

All 100 patients will undergo a conventional clinical EEG, using 19 channels in 10-20 arrangement, at the Department of Neurophysiology at the WCFT. Participant visiting time will last approximately one hour.

Saliva

All 100 patients will have samples of unstimulated saliva collected by soaking a sponge swab in the mouth of each participant until the swab is saturated with saliva. The swab will be inserted into a collection tube. In the laboratory, the saliva sample will then be collected into an Eppendorf tube by squeezing the saturated swab using a syringe. The sample will be stored at -80 C freezer until assay.

Data analysis

Clinical MRI

Analysis of clinical MRI data will be performed in all 100 participants recruited. We will perform morphometric analysis of subcortical structures which we know are implicated in epileptic seizures. Analysis of the data will involve using stereology in conjunction with point counting[15,16] and an automated method of volumetry for 3DT1-weighted MRI data[17] to estimate the volume of the hippocampus, amygdala, thalamus, and basal ganglia in all patients.

Advanced MRI*

**analysis of advanced MRI data will only be performed in the 40 participants that are included in the exploratory sub-study*

(1) Thalamocortical. Preliminary data from our group has indicated that patients with newly diagnosed epilepsy who continue to experience seizures despite AED therapy have diffusion ketosis imaging (DKI) alterations of thalamic projections; we will apply the same DKI approaches in our prospective study. Mean DKI values will be obtained from spatially co-registered regions-of-interest (principally thalamocortical regions) in standard space. We will also apply diffusion and resting-state functional MRI independent component analysis techniques using FSL's MELODIC toolbox (http://fsl.fmrib.ox.ac.uk/fsl/fslwiki/MELODIC) and in-house Matlab scripts to identify abnormal structural and functional thalamocortical connectivity in patients relative to controls[18-20]. (2) White matter tracts. Our recent publications have indicated that analysis of white matter tract diffusion has significance for predicting postsurgical seizure outcome in patients with chronic focal epilepsy and that DKI is more sensitive to tract pathology than diffusion tensor imaging in epilepsy[21-23]. As white matter tracts constitute the structural connections within brain networks,

For peer review only - http://bmjopen.bmj.com/site/about/guidelines.xhtml

we will determine DKI properties along the length of multi-lobar white matter tract bundles, using our recently reported methods[23,24]. (3) Connectome. The development of whole brain connectomes from diffusion MRI data have led to successful data-driven approaches to predict surgical responsiveness in patients with refractory focal epilepsy from members of our group[25-28]. Connectome approaches also support the association between postoperative seizure control and thalamocortical connectivity[25]. Similar methods have been applied to resting-state functional MRI data to model functional connectome alterations in chronic focal epilepsy[29]. As per our recent connectomic studies, whole brain structural connectomes will be generated for each participant using T1-weighted and DKI data. T1-weighted data will be parcellated into multiple regions of interest (ROI; or nodes) using Freesurfer software (http://freesurfer.net). Structural connectivity between nodes will be determined using FSL's diffusion toolbox (http://fsl.fmrib.ox.ac.uk/fsl/fslwiki/FDT) for probabilistic fibre tracking applied to diffusion MRI. Structural connectomes will be generated using the Connectome Mapping Toolkit (http://www.connectome.ch). We will use graph theory to determine global and regional network configuration. Global network "small worldness" will be assessed, representing the ratio between average nodal clustering coefficients and as network efficiency. Regional clustering coefficient, efficiency and centrality will also be calculated for key brain areas associated with seizure onset and propagation, such as thalamocortical and limbic networks.

EEG

The resting-state EEG activity of all 100 patients recruited will be identified by a trained clinical EEG professional. Nodes in EEG networks will be defined as electrodes, and a range of measures of interdependence between electrodes will be explored. We will apply computer models of network dynamics to resting-state EEG data (Schmidt et al 2016).

Blood samples

Samples from all 100 patients recruited will be analysed for inflammatory markers, namely HMGB1. Inflammatory marker and HMGB1 expression analysis will be undertaken by ELISA.

Saliva

Saliva samples will be analysed from all 100 patients to assess for concentration levels of circulating inflammatory markers including cytokine profile

Statistical analysis

To explore the utility of imaging, EEG and circulating inflammatory markers a series of univariable Cox regression models and a multivariable Cox regression model will be fitted for the outcome of time to next seizure following the index event, building on previous models developed with the MESS data[30]. For model development at least 10 events per candidate predictor variable is advocated[31]. With 100 patients and an assumed 2 year seizure recurrence rate of 51%[13], we would be able to include up to 5 predictor levels. Backward selection, using all candidate factors (imaging, EEG and HMGB1),according to Akaike's Information Criterion[32] will be used to determine the parsimonious model. Bootstrap resampling with 1000 replications will be used to adjust the developed model for optimism. Accuracy of model predictions will be explored using discrimination (Harrell's c-statistic) and calibration (calibration slope) in the data used for model development (MESS data) both before and after bootstrap resampling. External validation of the optimism-adjusted model will be evaluated via Harrell's c-statistic and calibration plots. The data for the external validation is the 100 participants prospectively recruited for this study.

Patient and Public Involvement

The development of the research question and outcome measures used in this study have been informed by close collaboration with local and national epilepsy charities. Patient groups were able to be consulted on their priorities, experience and preferences. Patients and their families through the Mersey Regional Epilepsy Association are involved with all research activity by our group. Patients are involved in the recruitment and conduct of the whole study. Results will be disseminated once available through epilepsy research websites, social media channels and mailing lists.

For peer review only - http://bmjopen.bmj.com/site/about/guidelines.xhtml

ETHICS AND DISSEMINATION

Ethical Approval

The Chief Investigator has obtained approval from the North East – Tyne & Wear South Research Ethics Committee (20/NE/0078). The Chief Investigator will ensure a copy of the Trust R&D approval letter is available before accepting participants into the study. The study will be conducted in accordance with the recommendations for physicians involved in research on human subjects adopted by the 18th World Medical Assembly, Helsinki 1964 and later revisions.

Human Material

Blood and saliva samples will be taken with appropriate informed consent from all 100 of the prospectively recruited participants.

Samples will be taken in the Walton Centre NHS Foundation Trust, main outpatient building, on the day of MRI scan for the 60 participants who are having clinical MRI sequences only performed. For the 40 participants who will be having advanced brain imaging performed in addition to standard clinical MRI sequences, they will have their samples taken at the LiMRIC on the day of their MRI scan.

Blood and saliva samples will be stored in the Liverpool University Biobank (LUB) freezer room, which is housed in the Research Technology building within the LiMRIC. All samples will be stored and accessed in line with LUB standard operating procedures (SOP).

Samples of human material will be stored and archived in the Liverpool University Biobank for a period of five years following initiation of the study, after which time they will be safely and appropriately destroyed in line with standard practice. The five-year period was chosen to allow a reasonable time frame for further analysis, following ethical clearance, after completion of the study which is expected to be within three years. Explicit consent will be taken from patients for the prolonged storage of this material beyond the study end date, the material will be composed of unused serum and saliva samples in case of further unspecified analysis pending ethical approval. In line with LUB SOP, the University of Liverpool will act as the custodian of the stored serum and saliva samples and explicit consent of the CI will be sought for any application to use the samples for any other purpose other than for those of the study outlined in this protocol.

Anonymity and data governance

All EEG and MRI data will be pseudo-anonymised prior to being exported from the WCFT and LiMRIC respectively. Personal information will not be identifiable from the data. Names will be replaced with study ID numbers (PRAFUS001, PRAFUS002 etc), which can be backtracked to participant details using a key that is located with the chief investigator, AM, who is part of the primary care team.

Digital data will be transferred from the clinical site in which it has been acquired (WCFT) to the UoL (Clinical Sciences Building, Room 2.23) for analysis on a secure, password protected networked University of Liverpool computer.

This data will include MRI, EEG and clinical/demographic data. All data will be pseudo-anonymised from the point of extraction by staff at the clinical site and transferred to GA using a secure, encrypted external hard drive - at which point patients will sequentially be allocated study IDs as per the process outlined above.

All data in the study will be in a digital format as password protected computer files.

This will include data relating to acquired MRI or EEG information, demographic and clinical information collected both at baseline and follow up at the various time points. All of these files will be stored on a password protected University of Liverpool networked computer in pseudo-anonymised format from the point of acquisition using the naming format highlighted above.

For peer review only - http://bmjopen.bmj.com/site/about/guidelines.xhtml

Pseudo-anonymised digital data highlighted above will be archived on a secure, password protected networked University of Liverpool computer located on the second floor of the Clinical Sciences Building, Room 2.23 and will be stored for a period of 20 years following study completion. Following this time all data will be safely and appropriately deleted. Data will be kept on a secure server that offers specialised storage of many terabytes of data per project which can comfortably accommodate all the data files generated from the study.

Data monitoring committee

As the nature of this study was observational, it was deemed appropriate that a data monitoring committee would not be required.

Informed Consent Process

All participants will be provided with a research information pack (appendix 1) describing the nature and goals of the research, and study consent form (appendix 2), which must be completed, signed and dated. We will not recruit participants who lack capacity to provide informed consent (e.g. those with intellectual disability or dementia). All participants will have the opportunity to discuss all aspects of the study with the Investigators. Participants will have as long as they require to consider their decision to volunteer for the research or not. The investigators contact details will be provided in the information pack. Participants are free to withdraw from the study at any time.

Confidentiality

The Chief Investigator will preserve the confidentiality of participants taking part in the study and is registered under the Data Protection Act.

Indemnity

The University of Liverpool holds Indemnity and insurance cover with Marsh UK LTD, which apply to this study.

Sponsor

The University of Liverpool will act as the main Sponsor for this study. Delegated responsibilities will be assigned to the NHS trusts taking part in this study.

Audits

The study may be subject to inspection and audit by the University of Liverpool under their remit as sponsor and other regulatory bodies to ensure adherence to GCP and the NHS Research Governance Framework for Health and Social Care (2nd edition).

Modification of the Protocol

Any modifications to the protocol which may impact on the conduct of the study, potential benefit of the patient or may affect patient safety, including changes of study objectives, study design, patient population, sample sizes, study procedures, or significant administrative aspects will require a formal amendment to the protocol. Such amendment will be agreed upon by research team and approved by the REC prior to implementation and notified to the health authorities in accordance with local regulations.

Dissemination

The aim will be to publish the results in high-quality peer-reviewed journals and to present at national and international conferences. We will target epilepsy-specific events (e.g. European Congress for Epileptology, International Epilepsy Congress, International League Against Epilepsy (ILAE) UK Chapter, American Epilepsy Society).

For each publication, only members of the research team who made a significant intellectual contribution to each piece of work will be considered as an author. This is in line with journal protocol. All authors share responsibility for the contents of the submitted manuscript.

Final data set

For peer review only

The CI and members of the research team will have access to the final cleaned data set that will be stored in accordance with secure data management methods as highlighted earlier in this protocol.

ACKNOWLEDGEMENTS

The authors are thankful to members of staff in the Institution of Translational Medicine (ITM), UoL for internal peer review of the original grant proposal and study costings for the fellowship application. The authors acknowledge all investigators at the planned recruitment site (WCFT) and Dr Kumar Das (Department of Neuroradiology, WCFT), Dr Shubhabrata Biswas (Department of Neuroradiology, WCFT), Dr Surjit Lyons-Nandra (Department of Neurophysiology, WCFT) for their support with the study.

We are incredibly grateful for the feedback of the patients and their families in designing this study as well as the support of all recruited participants without whom this study would not be possible.

FOOTNOTES

Author Contributions: CdB, SSK and GA contributed to the design of the MRI procedures and prepared the protocol for publication. LB contributed to the statistical elements of the study and sample size calculations. PL, MR and MP contributed to EEG methodologies and analysis. GA, TM and SSK contributed to the overall design of the study, leading on the setup of data collection methods at recruitment sites. GA, TM, SSK and CdB contributed to the analysis plan. SB, KD, CdB and SSK devised clinical and research standard imaging analysis for the study. GA, TM and SSK conceived the study and led the development of the protocol.

All authors provided critical intellectual input as per ICMJE criteria to the manuscript and have approved the final version for publication.

Funding: This work is supported by the Association of British Neurologists (ABN) and Guarantors of Brain (GoB) via a clinical research training fellowship awarded to GA and sponsored by the UoL (6107).

Disclaimer: The trial sponsor and funders had no role in trial design; collection, management, analysis and interpretation of data; writing of the report; and the decision to submit the report for publication, nor will they have ultimate authority over any of these activities. The views and opinions expressed herein are those of the authors and do not necessarily reflect those of the UoL, ABN, GoB and NHS.

Data sharing statement: Technical appendix, statistical code, and dataset will be made freely available from an appropriate data repository.

Competing interests: None declared.

Word Count: 3986 words.

For peer review only - http://bmjopen.bmj.com/site/about/guidelines.xhtml

REFERENCES

1. Hauser, W. A., & Beghi, E. (2008). First seizure definitions and worldwide incidence and mortality. *Epilepsia*, *49*, 8–12. https://doi.org/10.1111/j.1528-1167.2008.01443.x

2. Rizvi, S., Ladino, L. D., Hernandez-Ronquillo, L., & Téllez-Zenteno, J. F. (2017). Epidemiology of early stages of epilepsy: Risk of seizure recurrence after a first seizure. *Seizure*, *49*, 46–53. https://doi.org/10.1016/j.seizure.2017.02.006

3. Berg, A. T., & Shinnar, S. (1991). The risk of seizure recurrence following a first unprovoked seizure: a quantitative review. *Neurology*, *41*(7), 965–972. http://www.ncbi.nlm.nih.gov/pubmed/2067659

4. Debicki, D. B. (2017). Electroencephalography after a single unprovoked seizure. *Seizure*, *49*, 69–73. https://doi.org/10.1016/j.seizure.2017.03.001

5. Marson, A., Jacoby, A., Johnson, A., Kim, L., Gamble, C., Chadwick, D., & Medical Research Council MESS Study Group. (2005). Immediate versus deferred antiepileptic drug treatment for early epilepsy and single seizures: a randomised controlled trial. *The Lancet*, *365*(9476), 2007–2013. https://doi.org/10.1016/S0140-6736(05)66694-9

6. Bonnett, L. J., Tudur-Smith, C., Williamson, P. R., & Marson, A. G. (2010). Risk of recurrence after a first seizure and implications for driving: Further analysis of the multicentre study of early epilepsy and single seizures. *BMJ (Online)*, *341*(7785), 1260. https://doi.org/10.1136/bmj.c6477

7. Kim, L. G., Johnson, T. L., Marson, A. G., Chadwick, D. W., & MRC MESS Study group. (2006). Prediction of risk of seizure recurrence after a single seizure and early epilepsy: further results from the MESS trial. *The Lancet Neurology*, *5*(4), 317–322. https://doi.org/10.1016/S1474-4422(06)70383-0

8. Keller, S. S., Richardson, M. P., Schoene-Bake, J. C., O'Muircheartaigh, J., Elkommos, S., Kreilkamp, B., Goh, Y. Y., Marson, A. G., Elger, C., & Weber, B. (2015). Thalamotemporal alteration and postoperative seizures in temporal lobe epilepsy. *Annals of Neurology*, *77*(5), 760–774. https://doi.org/10.1002/ana.24376

9. Keller, S. S., Glenn, G. R., Weber, B., Kreilkamp, B. A. K., Jensen, J. H., Helpern, J. A., Wagner, J., Barker, G. J., Richardson, M. P., & Bonilha, L. (2017). Preoperative automated fibre quantification predicts postoperative seizure outcome in temporal lobe epilepsy. *Brain*, *140*(1), 68–82. https://doi.org/10.1093/brain/aww280

10. Walker, L. E., Frigerio, F., Ravizza, T., Ricci, E., Tse, K., Jenkins, R. E., Sills, G. J., Jorgensen, A., Porcu, L., Thippeswamy, T., Alapirtti, T., Peltola, J., Brodie, M. J., Park, B. K., Marson, A. G., Antoine, D. J., Vezzani, A., & Pirmohamed, M. (2017). Molecular isoforms of high-mobility group box 1 are mechanistic biomarkers for epilepsy. *The Journal of Clinical Investigation*, *127*(6), 2118–2132. https://doi.org/10.1172/JCI92001

11. Schmidt, H., Woldman, W., Goodfellow, M., Chowdhury, F. A., Koutroumanidis, M., Jewell, S., Richardson, M. P., & Terry, J. R. (2016). A computational biomarker of idiopathic generalized epilepsy from resting state EEG. *Epilepsia*, *57*(10), e200–e204. https://doi.org/10.1111/epi.13481

12. Peduzzi, P., Concato, J., Feinstein, A. R., & Holford, T. R. (1995). Importance of events per independent variable in proportional hazards regression analysis II. Accuracy and precision of regression estimates. *Journal of Clinical Epidemiology*, *48*(12), 1503–1510. https://doi.org/10.1016/0895-4356(95)00048-8

13. First Seizure Trial Group. (1993). Randomized clinical trial on the efficacy of antiepileptic drugs in reducing the risk of relapse after a first unprovoked tonic-clonic seizure. *Neurology*, *43*, 478–483. https://doi.org/10.1016/0736-4679(93)90655-Q

14. Browne R. H. (1995). On the use of a pilot sample for sample size determination. *Statistics in medicine*, *14*(17), 1933–1940. https://doi.org/10.1002/sim.4780141709

15. Keller, S. S., Highley, J. R., Garcia-Finana, M., Sluming, V., Rezaie, R., & Roberts, N. (2007). Sulcal variability, stereological measurement and asymmetry of Broca's area on MR images. *Journal of anatomy*, *211*(4), 534–555. https://doi.org/10.1111/j.1469-7580.2007.00793.x

16. Keller, S. S., Gerdes, J. S., Mohammadi, S., Kellinghaus, C., Kugel, H., Deppe, K., Ringelstein, E. B., Evers, S., Schwindt, W., & Deppe, M. (2012). Volume estimation of the thalamus using freesurfer and stereology: consistency between methods. *Neuroinformatics*, *10*(4), 341–350. https://doi.org/10.1007/s12021-012-9147-0

For peer review only - http://bmjopen.bmj.com/site/about/guidelines.xhtml

17. Amann, M., Andělová, M., Pfister, A., Mueller-Lenke, N., Traud, S., Reinhardt, J., Magon, S., Bendfeldt, K., Kappos, L., Radue, E. W., Stippich, C., & Sprenger, T. (2014). Subcortical brain segmentation of two dimensional T1-weighted data sets with FMRIB's Integrated Registration and Segmentation Tool (FIRST). *NeuroImage. Clinical*, *7*, 43–52. https://doi.org/10.1016/j.nicl.2014.11.010

18. O'Muircheartaigh, J., Vollmar, C., Barker, G. J., Kumari, V., Symms, M. R., Thompson, P., Duncan, J. S., Koepp, M. J., & Richardson, M. P. (2011). Focal structural changes and cognitive dysfunction in juvenile myoclonic epilepsy. *Neurology*, *76*(1), 34–40. https://doi.org/10.1212/WNL.0b013e318203e93d

19. O'Muircheartaigh, J., Keller, S. S., Barker, G. J., & Richardson, M. P. (2015). White Matter Connectivity of the Thalamus Delineates the Functional Architecture of Competing Thalamocortical Systems. *Cerebral cortex (New York, N.Y. : 1991)*, *25*(11), 4477–4489. https://doi.org/10.1093/cercor/bhv063

20. Beckmann, C. F., & Smith, S. M. (2005). Tensorial extensions of independent component analysis for multisubject FMRI analysis. *NeuroImage*, *25*(1), 294–311. https://doi.org/10.1016/j.neuroimage.2004.10.043

21. Keller, S. S., Richardson, M. P., Schoene-Bake, J. C., O'Muircheartaigh, J., Elkommos, S., Kreilkamp, B., Goh, Y. Y., Marson, A. G., Elger, C., & Weber, B. (2015). Thalamotemporal alteration and postoperative seizures in temporal lobe epilepsy. *Annals of neurology*, *77*(5), 760–774. https://doi.org/10.1002/ana.24376

22. Keller, S. S., Glenn, G. R., Weber, B., Kreilkamp, B. A., Jensen, J. H., Helpern, J. A., Wagner, J., Barker, G. J., Richardson, M. P., & Bonilha, L. (2017). Preoperative automated fibre quantification predicts postoperative seizure outcome in temporal lobe epilepsy. *Brain : a journal of neurology*, *140*(1), 68–82. https://doi.org/10.1093/brain/aww280

23. Glenn, G. R., Jensen, J. H., Helpern, J. A., Spampinato, M. V., Kuzniecky, R., Keller, S. S., & Bonilha, L. (2016). Epilepsy-related cytoarchitectonic abnormalities along white matter pathways. *Journal of neurology, neurosurgery, and psychiatry*, *87*(9), 930–936. https://doi.org/10.1136/jnnp-2015-312980

24. Kreilkamp, B. A., Weber, B., Richardson, M. P., & Keller, S. S. (2017). Automated tractography in patients with temporal lobe epilepsy using TRActs Constrained by UnderLying Anatomy (TRACULA). *NeuroImage. Clinical*, *14*, 67–76. https://doi.org/10.1016/j.nicl.2017.01.003

25. Bonilha, L., Helpern, J. A., Sainju, R., Nesland, T., Edwards, J. C., Glazier, S. S., & Tabesh, A. (2013). Presurgical connectome and postsurgical seizure control in temporal lobe epilepsy. *Neurology*, *81*(19), 1704–1710. https://doi.org/10.1212/01.wnl.0000435306.95271.5f

26. Bonilha, L., Jensen, J. H., Baker, N., Breedlove, J., Nesland, T., Lin, J. J., Drane, D. L., Saindane, A. M., Binder, J. R., & Kuzniecky, R. I. (2015). The brain connectome as a personalized biomarker of seizure outcomes after temporal lobectomy. *Neurology*, *84*(18), 1846–1853. https://doi.org/10.1212/WNL.0000000000001548

27. Hutchings, F., Han, C. E., Keller, S. S., Weber, B., Taylor, P. N., & Kaiser, M. (2015). Predicting Surgery Targets in Temporal Lobe Epilepsy through Structural Connectome Based Simulations. *PLoS computational biology*, *11*(12), e1004642. https://doi.org/10.1371/journal.pcbi.1004642

28. Munsell, B. C., Wee, C. Y., Keller, S. S., Weber, B., Elger, C., da Silva, L. A., Nesland, T., Styner, M., Shen, D., & Bonilha, L. (2015). Evaluation of machine learning algorithms for treatment outcome prediction in patients with epilepsy based on structural connectome data. *NeuroImage*, *118*, 219–230. https://doi.org/10.1016/j.neuroimage.2015.06.008

29. Liu, M., Bernhardt, B. C., Hong, S. J., Caldairou, B., Bernasconi, A., & Bernasconi, N. (2016). The superficial white matter in temporal lobe epilepsy: a key link between structural and functional network disruptions. *Brain : a journal of neurology*, *139*(Pt 9), 2431–2440. https://doi.org/10.1093/brain/aww167

30. Bonnett, L. J., Tudur-Smith, C., Williamson, P. R., & Marson, A. G. (2010). Risk of recurrence after a first seizure and implications for driving: further analysis of the Multicentre study of early Epilepsy and Single Seizures. *BMJ (Clinical research ed.)*, *341*, c6477. https://doi.org/10.1136/bmj.c6477

31. Peduzzi, P., Concato, J., Feinstein, A. R., & Holford, T. R. (1995). Importance of events per independent variable in proportional hazards regression analysis. II. Accuracy and precision of regression estimates. *Journal of clinical epidemiology*, *48*(12), 1503–1510. https://doi.org/10.1016/0895-4356(95)00048-8

32. Sauerbrei, W. (1999). The Use of Resampling Methods to Simplify Regression Models in Medical

For peer review only - http://bmjopen.bmj.com/site/about/guidelines.xhtml

Statistics. *Journal of the Royal Statistical Society. Series C (Applied Statistics)*, *48*(3), 313–329. http://www.jstor.org/stable/2680827

LIST OF FIGURES

1 Flow chart showing the recruitment process

APPENDICES

Appendix 1 – Participant information sheet

Appendix 2 – Participant consent form

For peer review only

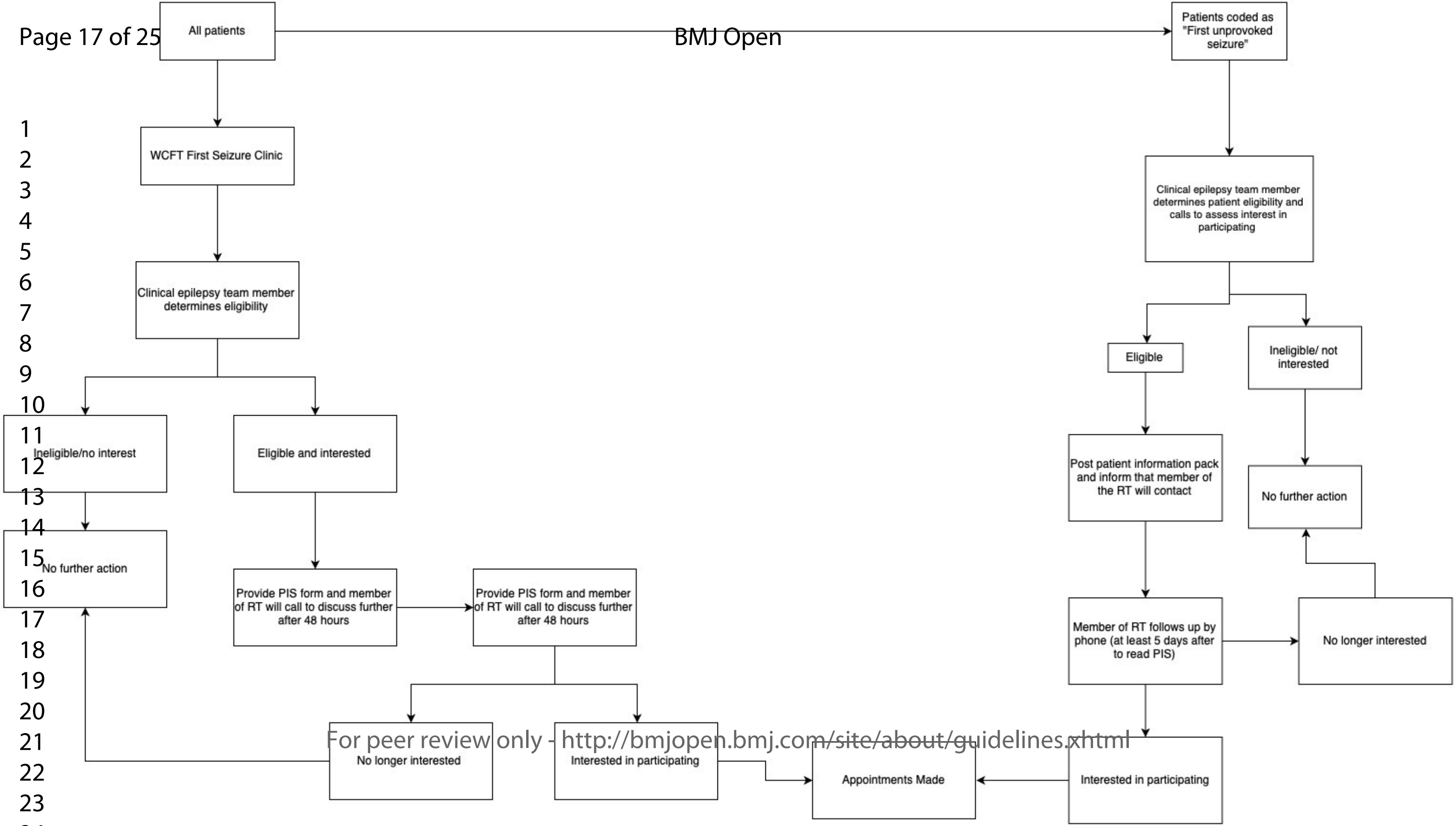

For peer review only - http://bmjopen.bmj.com/site/about/guidelines.xhtml

The Walton Centre NHS
NHS Foundation Trust

The Walton Centre NHS Foundation Trust
Lower Lane, Fazakerley
Liverpool, L9 7LJ, UK
Tel: 0151 525 3611
Fax: 0151 529 5500

Dr Guleed Adan
Clinical Research Fellow
2nd Floor, Neurological Science, Clinical Sciences Centre
Aintree University Hospital and The Walton Centre NHS Foundation Trusts
Lower Lane, Liverpool, L9 7LJ
Telephone: 0151 529 5943
Email: guleed@liverpool.ac.uk

PARTICIPANT INFORMATION SHEET FOR PATIENTS (PRAFUS-PIS-V4, 13/03/20)

Research study: Predicting recurrence after first unprovoked seizure (PRAFUS)

You are being invited to take part in a research study. Before you decide it is important for you to understand why the research is being done and what it will involve. Please take time to read the following information carefully. Talk to others about the study if you wish.

- Part 1 tells you the purpose of this study and what will happen to you if you take part.
- Part 2 gives you more detailed information about the conduct of the study.

Ask us if there is anything that is not clear or if you would like more information. Take time to decide whether you wish to take part.

PART 1

What is the purpose of the study?
People who have had a first unprovoked seizure may have an EEG and an MRI scan performed. At the moment, we find that many people with a first seizure often have a normal MRI and EEG. We think this is because we do not yet know the best way to carry out EEG and MRI scanning for people with first unprovoked seizure, and that the current use of scans may not be detailed enough. We have developed some new EEG and MRI scanning methods, and would like to try them out in people who have had a first unprovoked seizure. We hope that these new scans will provide us with a more detailed picture of the structure and function of the brain, which may be very important for people who have a first seizure so that we can better predict those that will go on to have further seizures and therefore develop epilepsy. We are hoping that our EEG and MRI scans can provide information on why some people have further seizures and others do not. We will also be taking blood and saliva samples that will be analysed for novel markers of inflammation that may help to identify whether increased levels of inflammation in the body is linked to the chance of having future seizures.

Why have I been chosen?
You have been chosen because we know you have been recently diagnosed with a first unprovoked seizure.

Do I have to take part?
No. It is entirely up to you to decide whether or not to take part. If you do, you will be given this information sheet to keep and be asked to sign a consent form. You are still free to withdraw at any time and without giving a reason. A decision to withdraw at any time, or a decision not to take part, will not affect the standard of clinical care you receive. If you express an interest in participating in this research, a member of the research team will contact you by your preferred method of correspondence.

What will happen to me if I take part?
We will send you an appointment for the following:

For peer review only

The Walton Centre NHS Foundation Trust
Lower Lane, Fazakerley
Liverpool, L9 7LJ, UK
Tel: 0151 525 3611
Fax: 0151 529 5500

(1) An MRI brain scan at LiMRIC or the Walton Centre. The MRI scan will last about 30 minutes and will involve you lying still and relaxing in the scanner. If your scan takes place at the LiMRIC, you will have some advanced brain scans performed that will involve some extra time to the standard clinical scans that are used currently. This will not compromise your clinical care and your scan will still be analysed and reported by a neuro-radiology consultant at the Walton Centre. Your report will still be returned to the neurology consultant that you are under the care of. A summary of the details of your MRI scan will be sent to you and to your GP as per the Walton Centre's standard clinical practice.

(2) After or before your MRI scan we will take a maximum of three blood tubes (containing nine millilitres each), which is the equivalent of an eggcup full of blood in total. We will also take a small salivary swab from the inside of the cheek. This will take around five minutes. Blood and saliva samples will be analysed and stored in a biobank (freezer) at the LiMRIC for a period of five years or until all of the sample has been used up, whichever is sooner.

(3) Finally, an EEG scan at the Walton Centre, where you will have already been seen by a specialist. This will last around one hour and will involve electrodes being placed on the surface of your head. You will be seated upright, awake and will be relaxed while measurements are taken. The EEG test will likely be on a different day to the MRI scan and blood sample.

There are no more scans or tests after this. A clinical member of the research team will contact you at four different time points after your first seizure: 6 months, 12 months, 18 months and 24 months. This will involve asking basic and brief questions about any further seizures you may have had.

What do I have to do?
All you need to do is sit and relax for the EEG scan and relax and lie still in the MRI scanner. A qualified health care professional will take a sample of your blood and saliva swab before or after your MRI scan.

What are the other possible disadvantages and risks of taking part?
The technique of MRI has been in use in medicine for about 30 years and has shown to be safe. It does not involve any radiation. In some people, there are times when it is not safe to be scanned. For example, in the first three months of pregnancy, or when there are surgical clips inside the brain, or if there is a heart pacemaker or Vagus Nerve Stimulator fitted. Furthermore, the scanner may get warm and noisy, and may not be suitable for sufferers of claustrophobia. However, rest assured, we will discuss with you thoroughly prior to the scan to identify any reasons why you should not have the scan. We will also thoroughly review with you on the day of scanning whether there is any possible risk for you. Earplugs are used to reduce the impact of scanner noise.

What are the possible benefits of taking part?
There may be no direct benefit to you from taking part. We hope that we will gain useful information predicting further seizures in people like yourself from these new scanning techniques, but we cannot be certain about this.

What if there is a problem?
Any complaint about the way you have been dealt with during the visit will be addressed. The detailed information on this is given in Part 2.

Will my taking part in the study be kept confidential?
Yes. All the information about your participation in this study will be kept confidential. The details are included in Part 2.

Contact Details:

For peer review only

The Walton Centre NHS
NHS Foundation Trust

The Walton Centre NHS Foundation Trust
Lower Lane, Fazakerley
Liverpool, L9 7LJ, UK
Tel: 0151 525 3611
Fax: 0151 529 5500

For further information, please contact any of the team running this research project:

Dr Guleed Adan
Clinical Research Fellow
2nd Floor, Neurological Science, Clinical Sciences Centre
Aintree University Hospital and The Walton Centre NHS Foundation Trusts
Lower Lane, Liverpool, L9 7LJ
Telephone: 0151 529 5943
Email: guleed@liverpool.ac.uk

Dr. Simon Keller
Senior Lecturer and Researcher in Neuroimaging
2nd Floor, Neurological Science
Clinical Sciences Centre
Aintree University Hospital and The Walton Centre
NHS Foundation Trusts
Lower Lane, Liverpool, L9 7LJ
Telephone: 07795617348
Email: simon.keller@liv.ac.uk

Professor Anthony Marson
Professor of Neurology
2nd Floor, Neurological Science
Clinical Sciences Centre
Aintree University Hospital and The Walton Centre
NHS Foundation Trusts
Lower Lane, Liverpool, L9 7LJ
Telephone: 0151 529 5770
Email: a.g.marson@liv.ac.uk

This completes Part 1 of the Information Sheet.
If the information in Part 1 has interested you and you are considering participation, please continue to read the additional information in Part 2 before making any decision.

For peer review only

The Walton Centre NHS
NHS Foundation Trust

The Walton Centre NHS Foundation Trust
Lower Lane, Fazakerley
Liverpool, L9 7LJ, UK
Tel: 0151 525 3611
Fax: 0151 529 5500

PART 2

What will happen if I don't want to carry on with the study?
You can withdraw from this research study at any time, even while you are having the tests done. This will not affect your treatment in any way. Even if you withdraw from the study, we would still like to use any information we might already have collected.

What if new information becomes available?
It is unlikely that any new information will become available while you are taking part in the study. Because we will be taking pictures of your brain with the MRI scan, occasionally we will have unexpected findings that none of us suspected. The pictures are reviewed by experienced doctors, called neuroradiologists who specialise in looking at pictures of brain. If there are any unexpected findings that need further tests, he/she will write to your GP or specialist. The doctor will then contact you if further tests are required. However, unexpected findings on MRI scans are rare.

What if there is a problem?
If you have a concern about any aspect of this study, you should ask to speak with the researchers who will do their best to answer your questions. If you remain unhappy and wish to complain formally, you can do this through the NHS Complaints Procedure. Details can be obtained from The Walton Centre NHS Foundation Trust:

Patient Experience Team
Sid Watkins Building
The Walton Centre
Lower Lane, Fazakerley, Liverpool, L9 7LJ
Tel: 0151 556 3090/3091
Email: patientexperienceteam@thewaltoncentre.nhs.uk

Further information on official complaints can be found here: https://www.thewaltoncentre.nhs.uk/362/comments-complaints-and-compliments.html

If something does go wrong and you are harmed during the research study there are no special compensation arrangements. If you are harmed and this is due to someone's negligence then you may have grounds for a legal action for compensation against the hospital, but you may have to pay your legal costs. The normal National Health Service complaints mechanisms will still be available to you.

Can I speak to anyone else?
If you would like to speak to someone not part of the research team about MRI scanning in general, please contact:

Professor Graham Kemp
Director, The Liverpool Magnetic Resonance Imaging Centre (LiMRIC)
Pembroke Place, Liverpool, L69 3GE
Telephone: 0151 794 5635; Email: gkemp@liverpool.ac.uk

For peer review only

The Walton Centre NHS
NHS Foundation Trust

The Walton Centre NHS Foundation Trust
Lower Lane, Fazakerley
Liverpool, L9 7LJ, UK
Tel: 0151 525 3611
Fax: 0151 529 5500

Who is organising and funding the research?
This research is being organised Dr Guleed Adan and is sponsored by The University of Liverpool (see below). A Clinical Research Training Fellowship awarded to Dr Adan, supervised by Prof. Marson and Dr. Keller is funding this research. Neither the research team nor your doctor receives any payment if you take part.

Will my taking part in this study be kept confidential?
Yes. We will collect information about you that could identify you personally (for example, because the information includes your name or date of birth). We will also collect information about you because we believe it might be relevant to understanding the research (e.g. information about your epilepsy, medicine, any previous scans and EEGs, dates and times of seizures near to the MRI scan). This information will be stored in "pseudo-anonymised" form (which means that your name, address and other personal details will be linked with the information we use in the research, but will not be directly accessible during the research) on computers owned by the hospital and on computers owned by the University of Liverpool. These computers will be securely controlled by the research team under the direct responsibility of Dr. Simon Keller and Professor Tony Marson, and no-one outside the team will have access to your information. We will use the information we collect to answer the questions relevant to this research project. Blood and saliva samples will be stored in anonymous form in dedicated research laboratories at the University of Liverpool until samples are used up.

The data that we collect from you (MRI scans, EEG scans, blood and saliva samples) will be kept for future research. All of this data will not include any identifiable information about you. The data may be made available to other researchers that work with the study team, but at no point is any information about who you are indicated or shared.

In the future, it is possible we might have new research questions that could be answered by looking at your information in new ways. We would seek approval from the Research Ethics Committee to use your information for new research projects. If the Research Ethics Committee believed we should contact you again to ask your permission to re-use your information, we will do so. The hospital has a duty to ensure research conducted here is of a high standard and auditors from the hospital may need to review any information we hold about you. The auditors will maintain the highest standards of confidentiality. Procedures for handling, processing, storage and destruction of your data are compliant with the Data Protection Act 1998.

General Data Protection Regulation
The University of Liverpool is the sponsor for this study based in the United Kingdom. We will be using information from you and your medical records in order to undertake this study and will act as the data controller for this study. This means that we are responsible for looking after your information and using it properly. The University of Liverpool will keep identifiable information about you for 20 years after the study has finished if you consent to be contacted about future studies. If you do not consent to being contacted about future relevant studies, your identifiable information will not be kept beyond 5 years after the study has finished.

Your rights to access, change or move your information are limited, as we need to manage your information in specific ways in order for the research to be reliable and accurate. If you withdraw from the study, we will keep the information about you that we have already obtained. To safeguard your rights, we will use the minimum personally-identifiable information possible.

You can find out more about how we use your information by contacting Dr Guleed Adan (or alternative team member). Our Data Protection Officer is Victoria Heath and you can contact them at V.Heath@liverpool.ac.uk.

The University of Liverpool will use your name, NHS number and contact details to contact you about the research study, and make sure that relevant information about the study is recorded for your care, and to

For peer review only

The Walton Centre NHS Foundation Trust
Lower Lane, Fazakerley
Liverpool, L9 7LJ, UK
Tel: 0151 525 3611
Fax: 0151 529 5500

oversee the quality of the study. Individuals from the University of Liverpool and regulatory organisations may look at your medical and research records to check the accuracy of the research study. The Walton Centre will pass these details to the University of Liverpool along with the information collected from you and your medical records. The only people in The University of Liverpool who will have access to information that identifies you will be people who need to contact you to arrange an appointment for the research study investigations or audit the data collection process. The people who analyse the information will not be able to identify you and will not be able to find out your name, NHS number or contact details.

When you agree to take part in a research study, the information about your health and care may be provided to researchers running other research studies in this organisation and in other organisations. These organisations may be universities, NHS organisations or companies involved in health and care research in this country or abroad. Your information will only be used by organisations and researchers to conduct research in accordance with the UK Policy Framework for Health and Social Care Research (https://www.hra.nhs.uk/planning-and-improving-research/policies-standards-legislation/uk-policy-framework-health-social-care-research/).

This information will not identify you and will not be combined with other information in a way that could identify you. The information will only be used for the purpose of health and care research and cannot be used to contact you or to affect your care. It will not be used to make decisions about future services available to you, such as insurance.

Involvement of your doctor
The doctor looking after you in the hospital will be aware of your participation in this research study.

What will happen to the results of the research study?
The scientific results of this research study will be published in scientific and medical journals and may be discussed at scientific meetings. You will not be personally identified in any way.

Who has reviewed the study?
North East - Tyne and Wear South Research Ethics Committee has reviewed this study and given a favourable ethical opinion for this research.

You will be given a copy of the information sheet and a copy of your signed consent form to keep.

Thank you for considering taking part in this research project and thank you for taking the time to read the information sheets.

For peer review only - http://bmjopen.bmj.com/site/about/guidelines.xhtml

The Walton Centre NHS
NHS Foundation Trust

The Walton Centre NHS Foundation Trust
Lower Lane, Fazakerley
Liverpool, L9 7LJ, UK
Tel: 0151 525 3611
Fax: 0151 529 5500

CONSENT FORM

Short title of Project: Predicting recurrence after first unprovoked seizure (PRAFUS)
Version 2=3: PRAFUS-CON-V3; 13/03/2020
IRAS Project ID: 279362**;** Research ethics committee ID:

Name of Chief Investigator: Prof. Tony Marson

	Please initial box
1. I confirm that I have read and understand the participant information sheet dated 13/03/20 (Version 4: **PRAFUS-PIS-V4**) for the above study. I have had the opportunity to consider the information, ask questions and have had these answered satisfactorily.	☐
2. I understand that my participation is voluntary and that I am free to withdraw at any time, without giving any reason, without my medical care or legal rights being affected.	☐
3. I understand that relevant sections of any of my medical notes and data collected during the study may be looked at by responsible individuals from regulatory authorities or from the NHS Trust, where it is relevant to my taking part in this research. I give permission for these individuals to have access to my records.	☐
4. I agree for a blood and saliva sample to be taken and used for future research. I understand that blood and saliva samples will be stored in anonymous form in dedicated research laboratories at the University of Liverpool for a period of five years or until samples are depleted, whichever is sooner.	☐
5. I agree for the research team to share anonymised data collected in this study with other researchers; I understand that I will not be identified in anyway.	☐
6. I agree to be contacted by a clinical member of the research team at 6, 12, 18 and 24 months after my MRI scan by telephone who will ask me brief questions about my seizures and medication.	☐
7. I agree that I may be allocated to have advanced MRI brain scans to be performed at the University of Liverpool, which will include some additional scanning (sequences) to the standard clinical scans that would have otherwise been performed at the Walton Centre. I understand that my clinical care will not be compromised should I agree to this.	☐

When completed, 1 for patient; 1 for researcher site file; 1 (original) to be kept in medical notes

For peer review only

The Walton Centre NHS
NHS Foundation Trust

The Walton Centre NHS Foundation Trust
Lower Lane, Fazakerley
Liverpool, L9 7LJ, UK
Tel: 0151 525 3611
Fax: 0151 529 5500

8. I agree to be contacted in the future should another scanning study become available. ☐

Preferred method of contact:

Address	Email
Home telephone	Mobile telephone

9. I agree to take part in the above study. ☐

Name of Participant	Date	Signature
Name of legal guardian or parent<br>***(if participant is under 18)***	Date	Signature
Name of Person taking consent<br>(if different from researcher)	Date	Signature
Researcher	Date	Signature

When completed, 1 for patient; 1 for researcher site file; 1 (original) to be kept in medical notes

For peer review only
